# Novel Insights into DNA Methylation Features in Spermatozoa: Stability and Peculiarities

**DOI:** 10.1371/journal.pone.0044479

**Published:** 2012-10-02

**Authors:** Csilla Krausz, Juan Sandoval, Sergi Sayols, Chiara Chianese, Claudia Giachini, Holger Heyn, Manel Esteller

**Affiliations:** 1 Department of Clinical Physiopathology, Andrology Unit, University of Florence, Florence, Italy; 2 Fundacio Puigvert, Barcelona, Catalonia, Spain; 3 Cancer Epigenetics and Biology Program (PEBC), Bellvitge Biomedical Research Institute (IDIBELL), Barcelona, Catalonia, Spain; 4 Department of Physiological Sciences II, School of Medicine, University of Barcelona, Barcelona, Catalonia, Spain; 5 Institucio Catalana de Recerca i Estudis Avançats (ICREA), Barcelona, Catalonia, Spain; Clermont-Ferrand Univ., France

## Abstract

Data about the entire sperm DNA methylome are limited to two sperm donors whereas studies dealing with a greater number of subjects focused only on a few genes or were based on low resolution arrays. This implies that information about what we can consider as a normal sperm DNA methylome and whether it is stable among different normozoospermic individuals is still missing. The definition of the DNA methylation profile of normozoospermic men, the entity of inter-individual variability and the epigenetic characterization of quality-fractioned sperm subpopulations in the same subject (intra-individual variability) are relevant for a better understanding of pathological conditions. We addressed these questions by using the high resolution Infinium 450K methylation array and compared normal sperm DNA methylomes against somatic and cancer cells. Our study, based on the largest number of subjects (n = 8) ever considered for such a large number of CpGs (n = 487,517), provided clear evidence for i) a highly conserved DNA methylation profile among normozoospermic subjects; ii) a stable sperm DNA methylation pattern in different quality-fractioned sperm populations of the same individual. The latter finding is particularly relevant if we consider that different quality fractioned sperm subpopulations show differences in their structural features, metabolic and genomic profiles. We demonstrate, for the first time, that DNA methylation in normozoospermic men remains highly uniform regardless the quality of sperm subpopulations. In addition, our analysis provided both confirmatory and novel data concerning the sperm DNA methylome, including its peculiar features in respect to somatic and cancer cells. Our description about a highly polarized sperm DNA methylation profile, the clearly distinct genomic and functional organization of hypo- versus hypermethylated loci as well as the association of histone-enriched hypomethylated loci with embryonic development, which we now extended also to hypomethylated piRNAs-linked genes, provides solid basis for future basic and clinical research.

## Introduction

Human spermatogenesis is an outstandingly complex biological process which requires the concerted action of several thousands of genes [1]. An interesting feature of this biological process is the extremely large inter-individual variability of sperm production in healthy fertile men. The entity of this variation is well illustrated by a large recent study, reporting that total sperm number in the so called normal range (defined as 5^th^ -95^th^ percentile), varies from 40 millions to several hundred millions [2]. While a few genetic variants have been studied in relation to spermatogenic efficiency in normozoospermic men [3]–[6], the epigenetic aspects of such variations in the normozoospermic range is completely unexplored.

Apart from the large inter-individual variability of the above mentioned quantitative traits of spermatogenesis, semen of normozoospermic men contains a qualitatively (in terms of motility and morphology) heterogeneous sperm population. With the advent and diffusion of assisted reproductive techniques, a number of sperm selection methods have been developed in order to obtain sperm subpopulations enriched with highly motile and morphologically normal spermatozoa to be used for *in vivo* or *in vitro* insemination. The rationale behind selection is mainly related to a predicted higher functional competency and a higher genomic integrity of selected spermatozoa. Interestingly enough, despite the same testicular environment, biochemical markers [7], [8] as well as DNA integrity [9]–[12] show differences in distinct sperm fractions belonging to the same individual. It is still unknown whether these fractions also show differences in their methylation level.

Given that epigenetic signals such as DNA methylation and histone modifications are crucial for the proper functioning of the genome, phenotypic differences in sperm production (quantitative as well as qualitative traits) at both inter- and intra-individual level may also be due to an epigenetic variation. This hypothesis seems to be plausible if we consider that the epigenome of mature spermatozoa mirrors a series of sequential epigenetic reprogramming events (demethylation and *de novo* methylation) which may generate substantial epigenetic variability. The sole study addressing the question about intra- and inter-individual DNA methylation changes in normozoospermic men was based on a 12,198-feature CpG island microarray [13]. The authors reported significant variations for 6 genes both at the intra- and inter-individual level, concluding that epigenetic variations may contribute to the variable semen phenotype. On the other hand, a limited inter-individual variability in DNA methylation was observed in two recent studies comparing two sperm donors by using a methylated DNA immunoprecipitation (MeDIP) procedure and promoter arrays [14] and a genome-wide shotgun bisulfite sequencing [15].

With respect to intra-individual variability of epigenetic marks in quality-fractioned sperm populations from normozoospermic and oligozoospermic men, data are available only for promoter CpG islands of two spermatogenesis candidate genes, *DAZ* and *DAZL* [16]. In this study, significant differences in the *DAZL* promoter methylation were observed between normal and defective germ cell fractions from the same individual. Other evidences for a potential association of DNA methylation defects and impaired sperm quality derives from studies based on the comparison of men with different sperm parameters including subjects with abnormally low sperm motility/morphology and sperm number [16]–[25].

Given the paucity of data on intra- and inter-individual variability of sperm DNA methylation, we aimed to provide a detailed description based on the analysis of a total of 487,317 CpG sites. Our first question was whether different quality-fractioned sperm populations deriving from the same individual displayed differences at the DNA methylation level i.e whether “good” and “poor” quality spermatozoa differ not only in their metabolic markers and genome integrity but also in their methylation status. Our second aim was to assess the level of inter-individual variability by comparing the genome-wide methylation profiles of whole sperm populations and quality-fractioned sperm subpopulations of different normozoospermic subjects.

Finally, we aimed to get further insights into the sperm DNA methylome through the investigation on loci with “variable” and “conserved” DNA methylation levels between individuals and their relationship with chromatin modifications. In addition, in this part of the study, we focused on a singular topic, not addressed by others until now, that concerns the sperm methylation status of piRNAs (PIWI-interacting RNAs). This peculiar class of small non coding RNAs are specifically expressed in the testis and seem to be involved in the maintenance of genomic stability and germ cell function through the silencing, via DNA methylation, of mobile genetic elements such as transposons (reviewed in Aravin et al. [26]). In fact, knock-out mice models for the proteins involved in the piRNA biogenesis (MIWI, MILI, MIWI2) revealed a restoration of transposon activity, which is thought to be the cause of the observed sterility due to meiotic arrest [27], [28]. However, given that piRNAs have recently been identified also in human cancer cells and somatic cells, it has been proposed that piRNAs regulate gene expression more broadly than previously predicted (for review see Juliano et al. [29] and Siddiqi and Matushansky [29], [30]. In order to provide new insights into this largely unexplored topic, we investigated the piRNAs methylation status in spermatozoa and performed a comparative analysis with a differentiated somatic cell type (B cell) and a colorectal cancer cell line (HCT-116).

Our study, based on the largest genome-wide DNA methylation analysis available to date in a group of normozoospermic men, allowed us to both define the “normal” sperm DNA methylome with its peculiar features and discover a potential new role for sperm piRNAs in embryonic development.

## Materials and Methods

### Subjects

Ethics statement: All participating subject signed an informed consent and the project has been approved by the local Ethical Committee of the University Hospital Careggi.

Eight healthy normozoospermic individuals of Italian origin belonging to the upper normal range of sperm number were analyzed in this study. Sperm parameters, age and relevant phenotypic information are reported in Table S1. Considerable care was taken for the selection of subjects in order to provide a homogeneous group in terms of life style factors, age, BMI and semen characteristics. Special attention was paid in selecting only semen samples devoid of contaminating somatic cells in their ejaculate. The absence of leucocytes or uroepithelial cells was assessed by scoring 5 stained slides at the light microscope in all 8 samples. The purity of the swim-down fraction deriving from contaminating cells was documented by checking additional 5 slides at light microscopy. This procedure based on a two-step purity check granted a biologically irrelevant, if any, contamination in both whole semen and the swim-down fractions.

Three aliquots were obtained from each individual corresponding to: 1) whole sperm population after 1 hour from semen collection; 2) swim-up fraction; 3) swim-down fraction. For sample EC7, the swim-down fraction has been excluded due to DNA degradation. For 3 samples whole, semen at 2 Sperm DNA methylation profile largely hours (corresponding to the time at which the swim-up procedure ends) were also available for the comparison with the other fractions.

### Sperm selection

Whole semen has been centrifuged on a 25% Percoll gradient (20 minutes) before the standard swim-up separation technique. Although much care was taken for the selection of samples in terms of lack of contaminating cells, this preliminary step further ensured the purity of the sperm population. The swim-up procedure allows spermatozoa with progressive motility to “swim up” into the culture medium while hypomotile/immotile spermatozoa remain behind. The upper fraction is denominated “Up”, whereas the fraction containing hypo/immotile spermatozoa is indicated in this manuscript as “Down or Dn”.

### Sperm DNA extraction

Sperm DNA was extracted with an user-developed version of the QIAamp® DNeasy&Tissue Kit purification protocol. Fresh washed (in PBS) sperm was incubated 1∶1 with a lysis buffer containing 20 mM TrisCl (pH 8), 20mM EDTA, 200 mM NaCl and 4% SDS, supplemented prior to use with 100 mM DTT and 250 ug/ml Proteinase K. Incubation was performed for 4 hours at 55°C with frequent vortexing. Prior to processing in the columns, 200 ul of absolute ethanol and 200 ul of the kit-provided lysis buffer were added to the samples. Then, purification was performed according to kit instructions.

### Microarray-based DNA methylation analysis

DNA was quantified by Quant-iT™ PicoGreen dsDNA Reagent (Invitrogen) and the integrity was analyzed in a 1.3% agarose gel. Bisulfite conversion of 600 ng of each sample was performed according to the manufacturer's recommendation for Illumina Infinium Assay. Effective bisulphite conversion was checked for three controls that were converted simultaneously with the samples. 4 µl of bisulfite converted DNA were used to hybridize on Infinium HumanMethylation 450 BeadChip, following Illumina Infinium HD Methylation protocol. Chip analysis was performed using Illumina HiScan SQ fluorescent scanner. The intensities of the images are extracted using GenomeStudio (2010.3) Methylation module (1.8.5) software. Methylation score of each CpG is represented as beta (β) value (β value <0.2 is considered as hypomethylated, >0.8 as hypermethylated). The 450K DNA Methylation array includes 485,764 cytosine positions of the human genome. From these cytosine sites, 482,421 positions (99.3%) are CpG dinucleotides, whilst only 3,343 sites (0.7%) correspond to CNG targets. Thus, from this point on we will use the term CpG, except when we refer specifically to putative CNG methylation. A general depiction of the 450K platform design, regarding functional genome distribution, CpG content and chromosome location, is reported in a previous validation study from our laboratory [31].

### Data filtering

The 450K DNA methylation array by Illumina is an established, highly reproducible method for DNA methylation detection and has been validated in two independent laboratories [31], [32].

Every *beta* value in the 450 K platform is accompanied by a detection p-value. We based filtering criteria on the basis of these p-values reported by the assay. We examined two aspects of filtering out probes and samples based on the detection p-values, selecting i) a threshold and ii) a cut-off. Previous analyses indicated that a threshold value of 0.01 allows a clear distinction to be made between reliable and unreliable *beta* values [31]. We selected the cut-off value as 10%. Following this criterion, we excluded all probes with detection p-values >0.01 in 10% or more of the samples and a total of 485,317 probes were included in the final analysis. We expect similar methylation level in neighbouring CpG sites given the strong correlation between CpG site methylation levels up to 150 bp.

### Statistical analysis

In order to identify differentially methylated CpG sites between different quality fractioned sperm populations, a non parametric test (Wilcoxon rank sum test) has been performed. Linear regression coefficient has been calculated (Spearman's rho) both for intra and inter-individual variability of methylation levels. For all comparisons of methylation levels between different subgroups Fischer exact test was performed.

For the estimation of the degree of epigenetic dissimilarity between individuals we measured the Euclidean distances between two samples using the following equation:
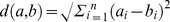
Where *a_i_* and *b_i_* represent the beta value for the i-essim CpG of samples “a” and “b”, and “n” the number of CpG sites selected.

In addition, to estimate the inter-individual variability of the methylation status in the promoters of the 6 genes previously described as highly variable, we calculated for each gene a 100,000 permutations test with the distances of the three groups, in order to obtain a random distribution of possible mean distances and get a p-value for the mean variability among individuals in a group (the area below the distribution curve).

For the estimation of enrichment in biological processes we performed a hypergeometric test on biological processes defined by Gene Ontology [33].

## Results

### Comparison of genome-wide DNA methylation level in different quality-fractioned sperm populations deriving from eight normozoospermic men

The ejaculate of a normozoospermic man contains a qualitatively heterogeneous sperm population (in terms of different motility, morphology, metabolic and genomic features). This part of the analysis focused on intra-individual variation and addressed the biological question whether there are significant differences in methylation profiling between the “up” (enriched with highly motile and morphologically normal spermatozoa) versus “down” (poorly motile/immotile and morphologically abnormal spermatozoa) semen fractions in each subject.

#### Analysis of intra-individual variation in 485,317 CpG loci

**Figure 1:**
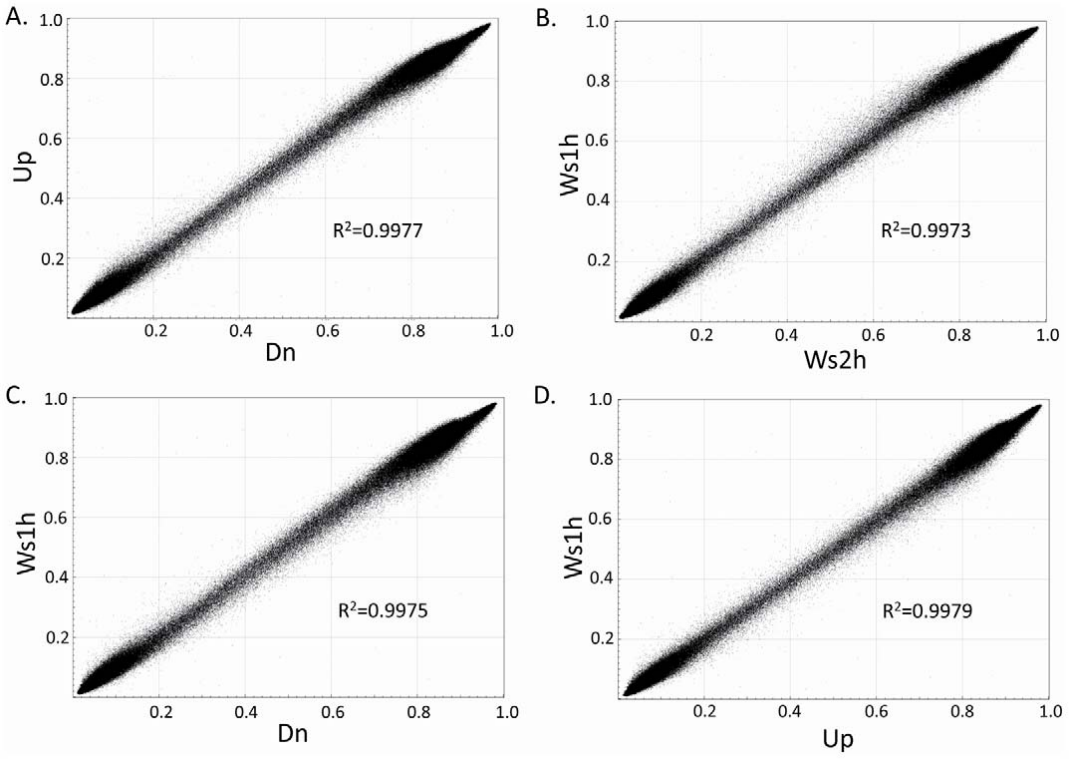
Scatter plots reporting CpGs methylation levels between different samples deriving from the swim-up sperm selection procedure in the same individual EC01: (A) swim-up (Up) sperm fraction versus swim-down (Dn) sperm fraction; (B) whole sperm population at1h (Ws 1 h) versus whole sperm population at 2 h (Ws 2h); (C) whole sperm population at 1h versus swim-down sperm fraction; (D) whole sperm population at1h versus swim-up sperm fraction. R^2^  = Pearson coefficient.

**Figure 2:**
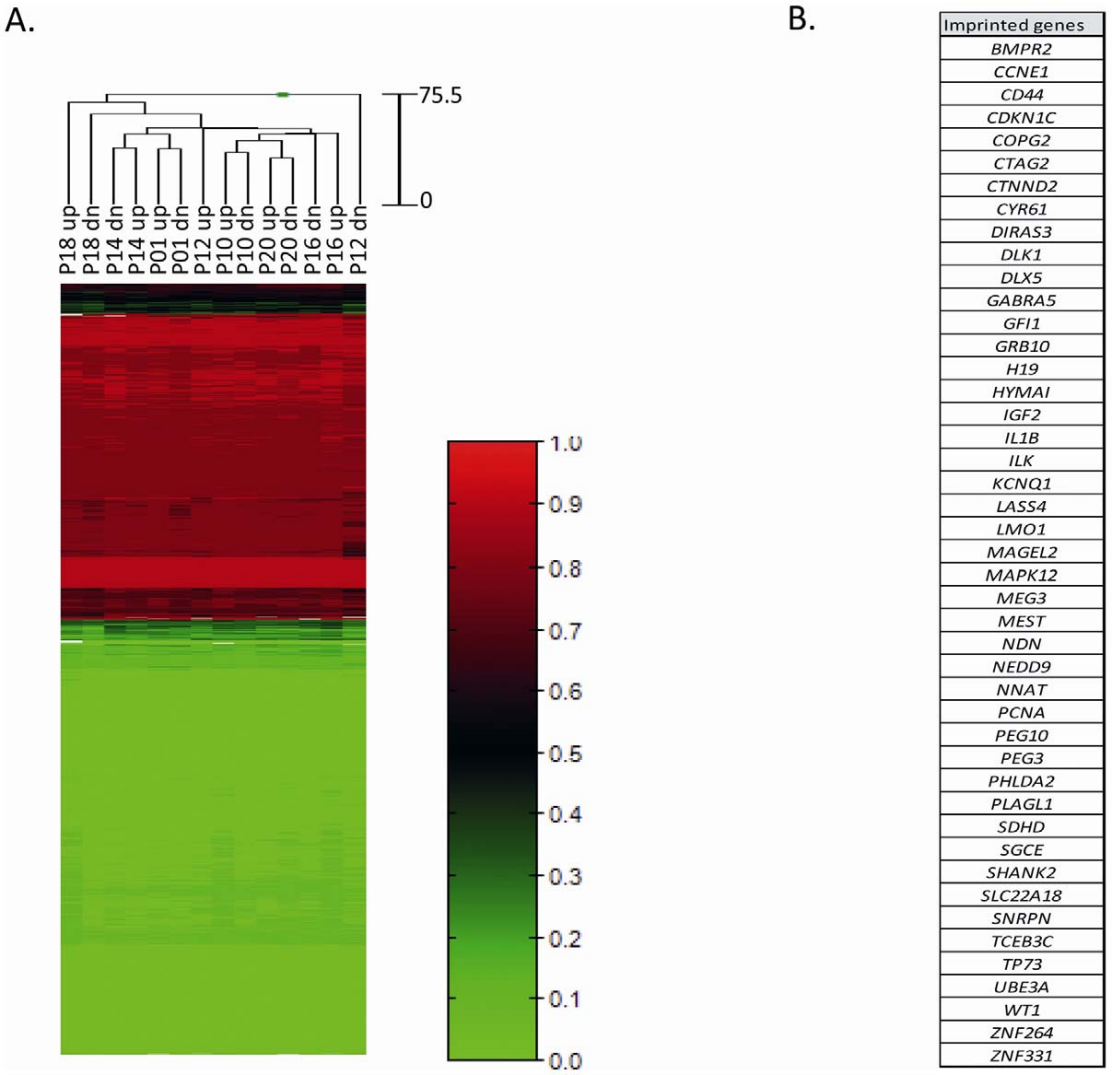
Heatmap displaying the methylation status of CpG loci (n = 2386) mapping in the promoters of 45 imprinted genes in relation to quality-fractioned sperm populations (i.e. swim-up “up” and swim- “dn” sperm fractions). A) The dendrograms above the heatmap show hierarchical clustering based on the methylation data alone. Sperm populations and CpG loci are represented by columns and rows, respectively. Each cell indicates the CpG methylation level for one site in each sample. Methylation levels are represented in the scale on the right side of the heatmap and are referred lowest to highest as green (0.0) to red (1.0). (B) List of the 45 imprinted gene available in the array.

**Figure 3:**
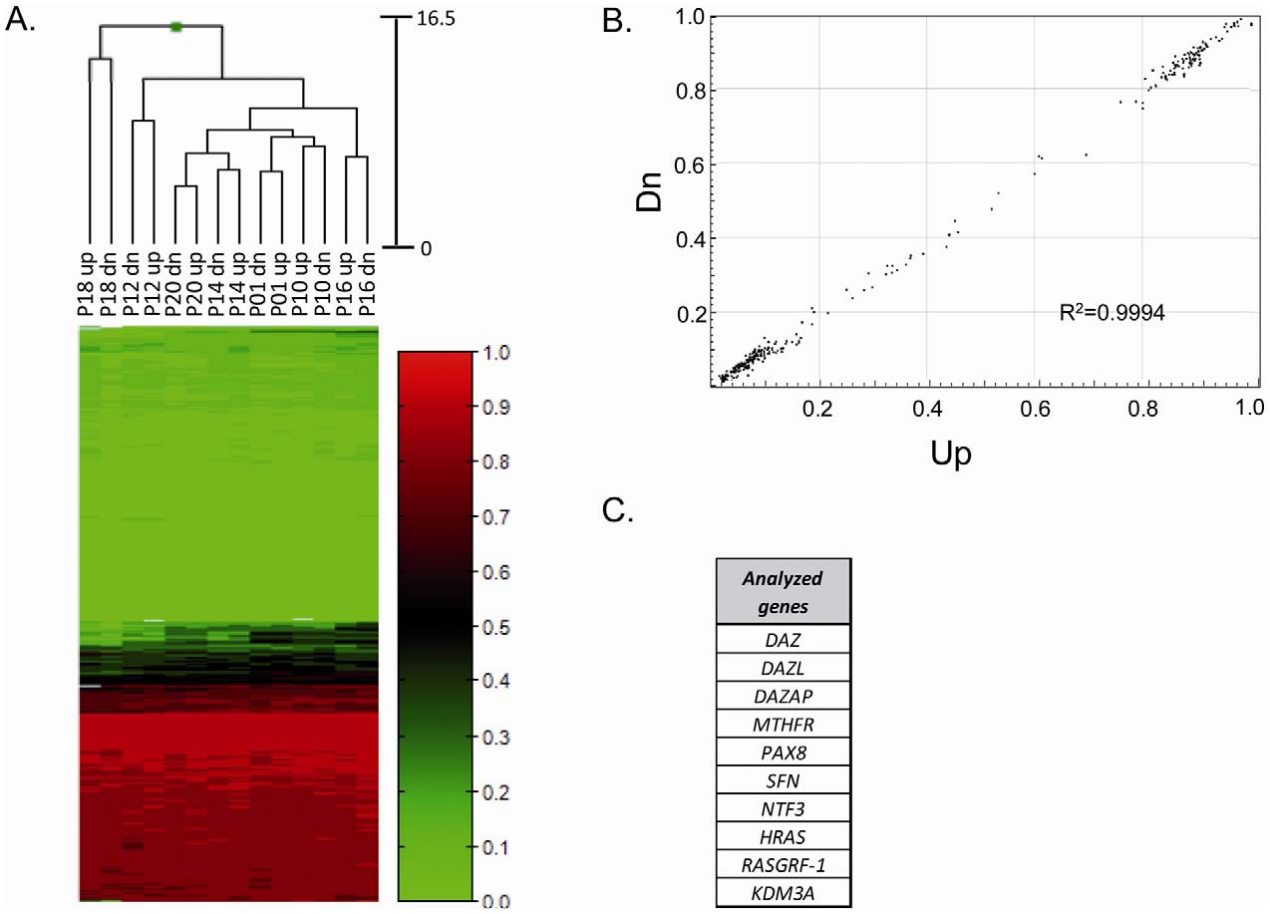
Heatmap displaying the methylation status of CpG loci (n = 297) mapping in 10 selected genes in relation to quality-fractioned sperm populations (i.e. swim-up “up” and swim-down “dn” sperm fractions). A) The dendrograms above the heatmap show hierarchical clustering based on the methylation data alone. Sperm populations and CpG loci are represented by columns and rows, respectively. Each cell indicates the CpG methylation level for one site in each sample. Methylation levels are represented in the scale on the right side of the heatmap and are referred lowest to highest as green (0.0) to red (1.0). B) Scatter plot reporting CpGs methylation levels between quality-fractioned sperm populations (Up vs Dn) among different individuals. R^2^ = Pearson coefficient. C) List of the 10 analyzed genes, selected because previously reported as differently methylated in infertile men compared to normozoospermic controls.

By performing linear regression analysis, we observed an extremely high correlation (Pearson correlation coefficient ranging from R^2^ = 0.9896 to R^2^ = 0.9982) between “good” and “poor” quality sperm suspensions in all subjects (Table S2A). A representative example is given for sample EC01 in Figure 1. Accordingly, unsupervised hierarchical cluster analysis of the two tested groups was unable to cluster the “up” and “down” fractions into two distinct groups. Similarly, no significant differences were observed following comparison of the methylation levels in the 485,317 CpG sites between different sperm fractions (“up” versus “down”, whole sperm population versus “down”, whole sperm population versus “up”). By comparing epigenetic distances between the “up” and “down” fractions of the same individual, we found no significant differences except for one sample (EC12) with p = 0.018. Interestingly, this sample showed the lowest sperm count among the 8 normozoospermic individuals. Separately, we analyzed the intra-individual variation in selected CpG loci previously reported to be associated with poor sperm quality. To begin with, a few imprinted loci were analyzed in previous studies in relationship with a wide range of infertile phenotypes (oligozoospermia, oligoasthenozoospermia, asthenozoospermia). All previous studies reported methylation changes in a portion of infertile men, suggesting that impaired sperm production may be associated with methylation defects. A total of 2,386 CpGs belonging to 45 imprinted genes are present on the 450K array (24) and we analyzed the methylation status of their promoter regions in the “up” and “down” fractions. Similarly, we investigated on 289 CpGs belonging to 10 genes (*DAZ, DAZL, DAZAP, HRAS, KDM3A, MTHFR, NTF3, PAX8, RASGRF1, SFN*) showing DNA methylation changes in infertile men compared to normozoospermic controls as well as in different quality-fractioned sperm populations (such as *DAZ* and *DAZL*). In all cases, a homogeneous methylation pattern was observed in the two fractions derived from the same individual and, accordingly, the two sperm fractions derived from all analyzed subjects did not cluster separately (Figure 2 and 3).

#### Assessment of inter-individual variability in genome-wide DNA methylation profile

**Figure 4:**
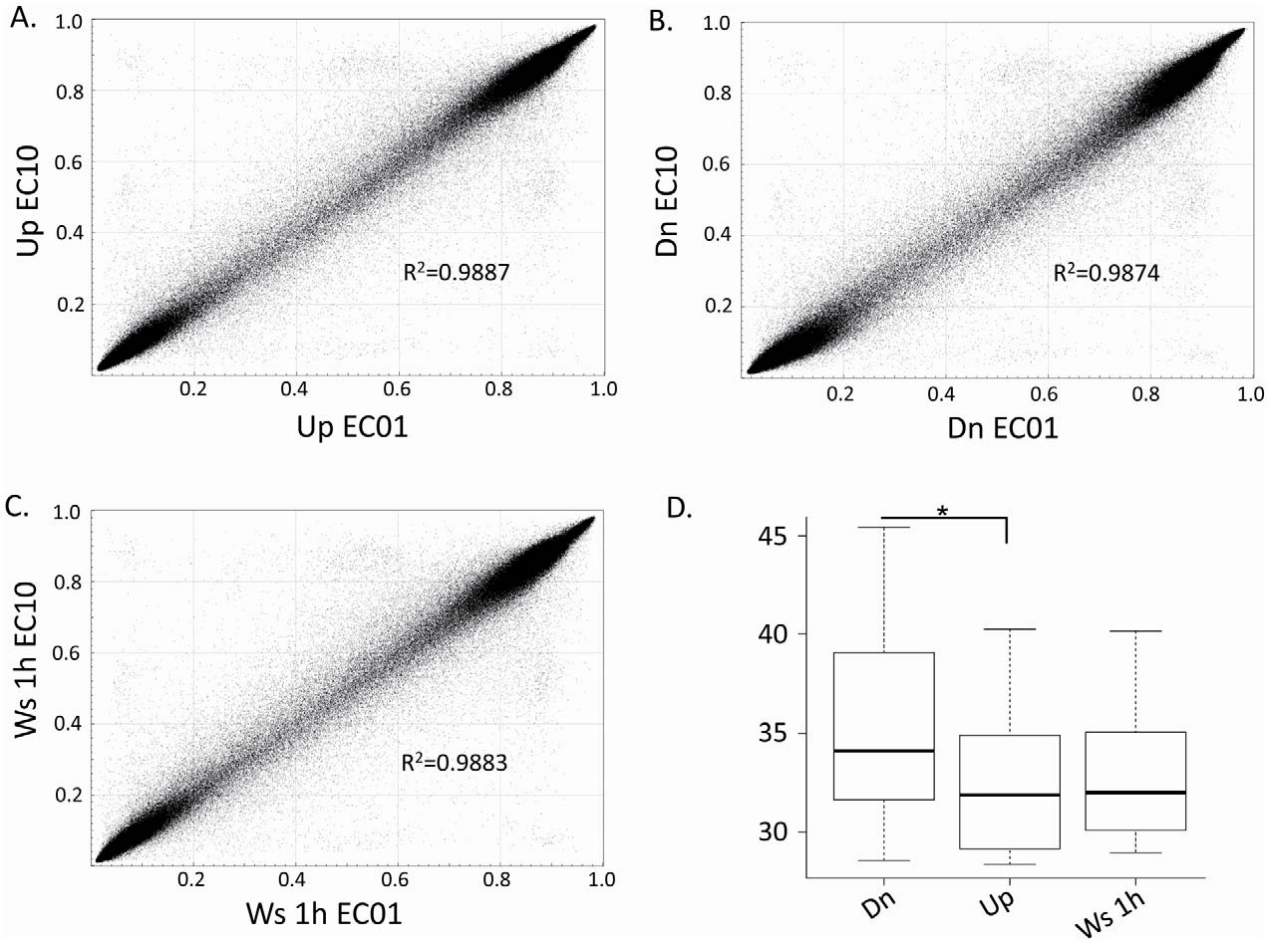
Representative scatter plots reporting CpG methylation levels between different individuals EC01 and EC10. (A) swim-up (Up) sperm samples; (B) swim-down (Dn) sperm samples; (C) whole sperm population at 1 h (Ws 1 h) samples; (D) Box plot representing the inter-individual variability of DNA methylation levels in total CpGs from the swim-up, swim-down and whole sperm population at1h samples. The median value is shown. * corresponds to p value = 0.0213; R2 = Pearson coefficient. The boxes describe the lower quartile (Q1, 25%), median (Q2, 50%) and the upper quartile (Q3, 75%); the whiskers extend 1.5 times the IQR from the box.

Although all subjects belonged to the upper normal range of sperm number, the whole semen fraction of each individual included a different proportion of “good” and “poor” quality spermatozoa. The most homogeneous sperm population containing the best quality spermatozoa was the “up” fraction. A slightly more pronounced inter-individual variability in DNA methylation profile has been observed compared to intra-individual variability between sperm fractions. However, the linear regression coefficients were always >0.98 (Table S2B). A representative scatter plot comparing two individuals is shown in Figure 4 A–C.

In order to further explore inter-individual variability, we analyzed each type of sperm fraction using two additional approaches: i) we quantified the number of CpGs showing a standard deviation >0.2 in the methylation level (*beta* value) between individuals; ii) we measured the epigenetic distance (by the use of the Euclidean distance formula reported in materials and methods) between the methylation level of CpGs in different subjects. The number of differentially methylated loci i.e. showing a SD >0.2 between different subjects was 1,591 in the whole semen, 1,207 in the “down” fraction and 1,675 in the “up” fraction. This implies that for all comparisons the number of CpGs above the established threshold level was very low, representing <0.3% of all loci tested. The GO analysis of genes related to the 1,675 differentially methylated sites did not show any germ cell specific function. (data not shown).

By performing the comparison of DNA methylation distances across individuals considering all 485,317 CpG sites, a significantly higher variability has been observed in the swim-down sperm fraction (p = 0.021) in respect to the swim-up fraction (Figure 4D). However, it is worth noticing that the coefficient of variation is still very low in the swim-down fraction, e.g. 14% which indicates that the maximum epigenetic distance between individuals was limited to 45, that is significantly lower than the maximum distance possible e.g. 696.(see Table S3).

#### Assessment of methylation level in six gene promoters previously reported as having the highest intra and inter-individual variation

**Figure 5:**
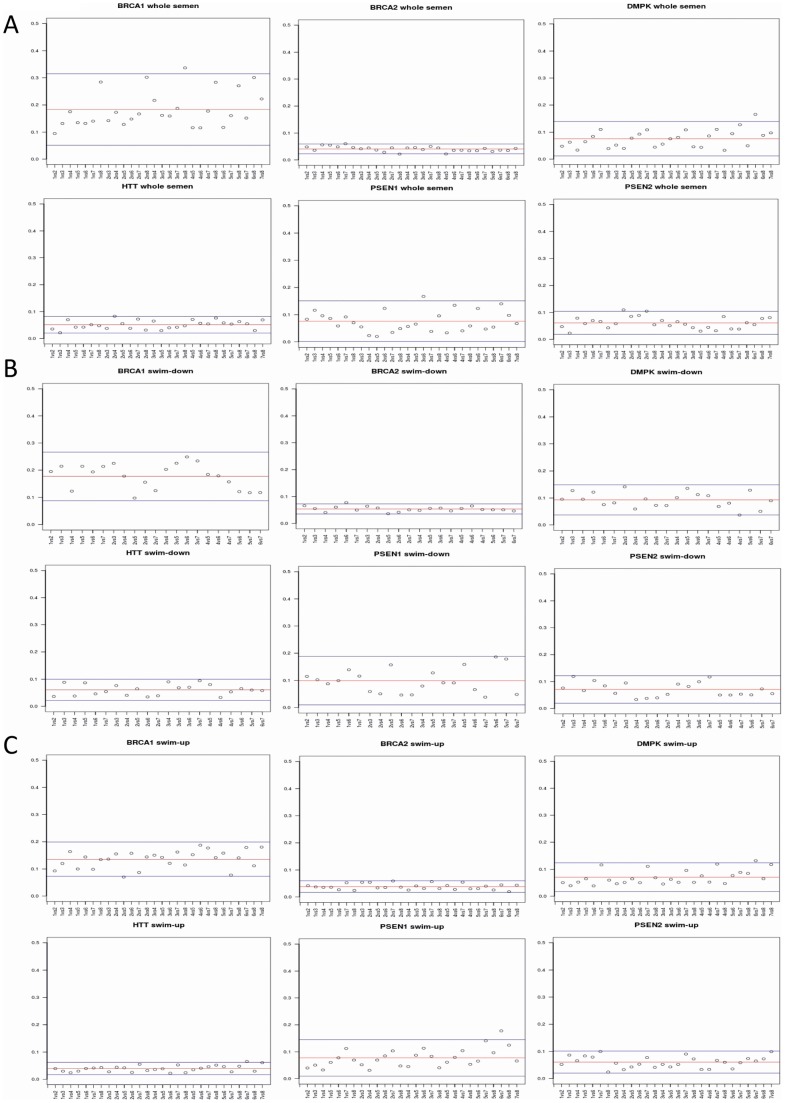
Intra-group epigenetic distances for the promoters of *BRCA1, BRCA2, HTT, DMPK1, PSEN1* and *PSEN2* genes. This distance represents the net dissimilarity of DNA methylation profiles between two sequences: the higher the distance, the more dissimilar are the compared samples. Different individuals were crossed with each other and Euclidean distances were calculated for beta-values of CpG sites as a marker of inter-individual variability in three different sperm subpopulations: A) Whole sperm population; B) swim-down and C) swim-up fractions. Numbers on the X axis indicate the identity of the pair-wise comparisons inside the experimental group: individuals EC01, EC07, EC10, EC12, EC14, EC16, EC18 and EC10 are numbered 1 to 8. Distance values are displayed on the Y axis. The top and bottom blue guidelines represent the 0.025 and 0.975 quartiles, while the red guideline represents the mean distance value.

Significant DNA methylation variations have been reported for promoters of the following 6 genes: *BRCA1, BRCA2, HTT (HD), DMPK (DM1), PSEN1,* PSEN2 by Flanagan et al. [13]. In order to evaluate the entity of inter-individual variability, we calculated Euclidean distances for the beta values of the CpG sites in the above gene promoters among individuals of the three groups (Whole semen, “Down” and “Up”), plots are shown in Figure 5A–C. In addition, by performing permutation test of the epigenetic distances we found significant inter-individual differences for 4 out of 6 genes (*HTT,DMPK, PSEN1, PSEN2*) in the swim-down sperm fraction (p values:2E-05; 0.00096; 0.00348; 0.02, respectively), while we observed no relevant variation in the swim-up fraction. In the whole sperm population sample, significance was reached only for *BRCA1* (0.01136).

### Sperm genome-wide methylome description and its comparison with differentiated somatic cells

#### Sperm DNA methylation profile: general features

Given that the swim-up fraction, being enriched in the best quality spermatozoa, is the one used for assisted reproductive techniques, we aimed to provide a detailed description of genome wide DNA methylation profile of these cells. The average DNA methylation level of the 485,317 CpG sites was 45% (median value 35%). However, an interesting feature of the sperm DNA methylome is the polarization of DNA methylation level towards the two extremes: 86% of all markers are either severely hypomethylated (<20%) or strongly hypermethylated (>80%). Intermediate methylation level (20–80%) was observed only for 14% of CpGs. We defined, in each subject, the number of hypomethylated and hypermethylated loci for the whole genome as well as for the sex chromosomes and autosomes, separately (Table 1). The coefficient of variation of DNA methylation levels was minimal between subjects for the hypomethylated loci (0.9%) and slightly higher for hypermethylated loci (2.8%), suggesting a highly conserved profile both for hypo and hypermethylated loci. Accordingly, we found 95.8% of all hypomethylated loci to be conserved between individuals (n = 220,300 CpGs), whereas in the hypermethylated loci the concordance was slightly lower, 86.1% (n = 161,542 CpGs).

**Table 1:** Description of CpGs in terms of number and methylation status in the swim-up sperm fraction and in B cells.

A.																	
	total					autosomes				X chromosome				Y chromosome			
	hypo			hyper		Hypo		hyper		hypo		hyper		hypo		hyper	
Patients	sperm		B cells	sperm	B cells	Sperm	B cells	sperm	B cells	sperm	B cells	sperm	B cells	sperm	B cells	sperm	B cells
**EC01**	228576		166537	190746	186229	221114	161321	188198	182663	7193	5113	2477	3414	274	113	71	152
**EC07**	231894		164595	191062	180334	224312	159359	188447	176829	7313	5098	2536	3351	276	110	79	154
**EC10**	228642			191830		221172		189232		7204		2521		271		77	
**EC12**	230315			187601		222808		185109		7251		2414		269		77	
**EC14**	230458			187734		222904		185222		7287		2438		272		74	
**EC16**	226746			187086		219322		184649		7166		2362		265		75	
**EC18**	233778			175270		226164		172984		7342		2220		276		64	
**EC20**	228732			188955		221292		186421		7180		2458		268		76	
**total CpG**	485317					473681				11220				416			
**mean**	229893	165566		187536	183282	222386	160340	185032	179746	7242	5106	2428	3383	271	112	74	153
**sd**	2209	1373		5263	4168	2142	1387	5163	4125	66	11	101	45	4	2	5	1
**percentage**	47.4	34.1		38.6	37.8	46.9	33.8	39.1	37.9	64.5	45.5	21.6	30.1	65.2	26.8	17.8	36.8

B.																
	“conserved” (total)				“conserved” (autosomes)				“conserved” (X chromosome)				“conserved” (Y chromosome)			
	hypo		hyper		Hypo		hyper		hypo		hyper		hypo		hyper	
	sperm	B cells	sperm	B cells	Sperm	B cells	sperm	B cells	sperm	B cells	sperm	B cells	sperm	B cells	sperm	B cells
**total CpG**	220300	160674	161542	173119	213088	155602	159532	169793	6958	4972	1953	3185	256	102	58	142
**percentage**	95.8	97.0	86.1	94.5	95.8	97.0	86.2	94.5	96.1	97.4	80.4	94.2	94.3	91.5	78.2	92.8

The number of hypomethylated and hypermethylated loci are indicated as total, autosomic, X chromosome and Y chromosome-linked. A) Sperm CpG numbers refer singularly to the eight normozoospermic men, while B cells CpG numbers belong to two different subjects. The mean CpG number ± DS and the percentage calculated in respect to the mean total CpG number for each group are reported in the middle panel; B) Number of CpGs conserved among individuals: the first raw reports the number of CpGs shared by individuals, while the second raw reports the percentage of conserved CpGs compared to the mean CpG number reported in panel A.

The separate analysis of autosomal (a total of 473,681 CpGs), X-linked (a total of 11,220 CpGs) and Y-linked CpGs (a total of 416 CpGs) revealed that X-linked loci are significantly more frequently hypomethylated than autosomal loci (64.5% versus 45.8%; p<2.2 xE-16), as was the case also for the Y–linked loci (65.2% versus 45.8%, p = 3.458xE-5) (Table 1). On the other hand, autosomal loci were significantly more frequently hypermethylated than X-linked loci (38.1% versus 21.6%; p<2.2xE-16). It is also worth noticing, that the highest percentage of “conserved” hypomethylated loci was found for the X-linked loci (96.1%).

#### Sperm DNA methylation profile: comparative analysis of regions with conserved hypo/hyper methylation and differentially methylated loci between individuals

**Figure 6:**
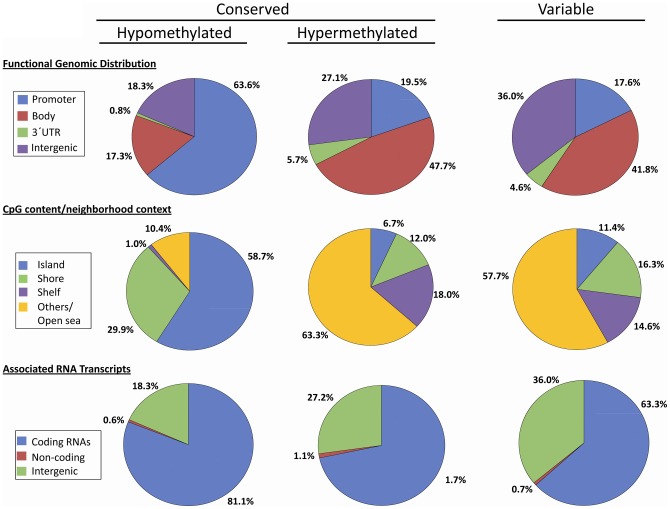
Sperm DNA methylation profile (swim-up sperm samples) according to i)functional genomic distribution; ii) CpG content/neighbourhood context and iii) associated RNA transcripts. (A) Distribution of hypomethylated (n = 220.300) and hypermethylated (n = 161.542) CpGs conserved within individuals B) Distribution of variable CpGs within individuals (n = 1674).

Subsequently, we identified loci displaying the same DNA methylation pattern in all subjects (“conserved” hypo or hyper) as well as loci showing different DNA methylation patterns (“variable” or “differentially methylated” loci). We analyzed the functional genomic distribution (promoter, body, 3′UTR, and intergenic), CpG content and neighborhood context. For the latter, we referred to: i) “island” as a DNA sequence (>200-bp window) with a GC content greater than 50% and an observed: expected CpG ratio of more than 0.6.; ii) “shore” as a sequence 0–2 Kb distant from the CpG island; iii) “shelf” as a sequence 2–4 Kb distant from the CpG island; iv) “open sea/others” as the remaining sequence. The methylation categories were also analyzed in relationship with genomic locations related to RNA transcription (coding, non-coding and intergenic). Sharp differences were observed between the “conserved” hypo and hyper-methylated loci according to the functional genomic distribution as well as for the CpG and neighborhood context (Figure 6). Among all “conserved” hypomethylated loci 63.6% belonged to promoters, whereas this percentage was significantly lower in the hypermethylated loci, 19.5% (p = 4,14E-05). A significant difference was observed also in the methylation status of gene body-linked CpGs which made up 47.7% of “conserved” hypermethylated loci and only a minority of the “conserved” hypomethylated loci (17.3%; p = 0.001357). Given the high prevalence of promoters in the hypomethylated sites, almost 90% of the hypomethylated CpGs correspond to islands and shores (58.7% and 29.9%, respectively). On the contrary, 80% of “conserved” hypermethylated sites are either in the CpG poor island shelves or in “open sea” regions (18% and 63.3%, respectively).

Differentially methylated loci (defined as >0.2 standard deviation between individuals) have been identified only for 0.3% of all analyzed CpGs. Interestingly, the percentage of “variable” regions were lower in X-linked loci (0.2%) and were completely absent in the 416 Y-linked loci. Intriguingly, the pattern of “variable” CpGs was more similar to the “conserved” hypermethylated loci than to the “conserved” hypomethylated ones, as a matter of fact the variation in DNA methylation between individuals is more pronounced in CpG-poor regions such as gene body, intergenic and “open sea” (Figure 6).

#### Gene Ontology analysis of “conserved” hypo and hypermethylated loci

Our next question was whether hypo and hypermethylated loci were linked to specific biological processes. By performing GO analysis, we found that the two methylation patterns are involved in completely distinct cellular processes (Table S4A). An outstandingly high association has been observed between hypomethylation and genes involved in metabolic and biosynthetic processes (among the first 20 significant associations, 11 are linked to metabolic and 5 to biosynthetic processes). On the contrary, hypermethylated sites, while associated with several different biological processes, did not show any association with metabolic and biosynthetic genes.

#### Analysis of DNA methylation levels in histone-enriched loci and gene ontology analysis

**Figure 7:**
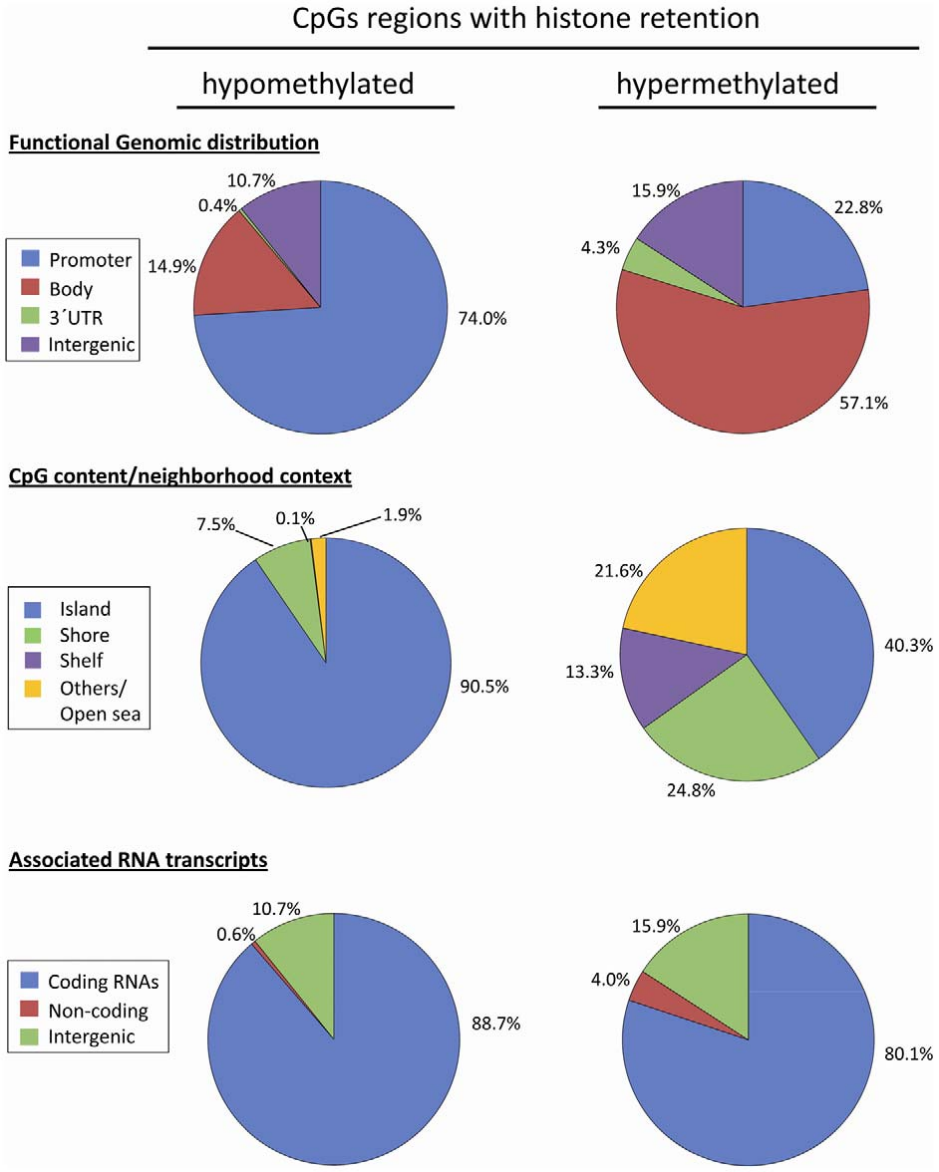
Sperm DNA methylation profile in histone-enriched regions according to i) functional genomic distribution; ii) CpG content/neighbourhood context; iii) associated RNA transcription. (A) Distribution of hypomethylated (n = 347) CpGs in swim-up sperm samples. (B) Distribution of hypermethylated (n = 30244) CpGs in swim-up sperm samples.

In a previous study, Hammoud et al.[14] defined the position of histone enriched loci in the sperm genome. We crossed our list of “conserved” hypo and hypermethylated loci with the list of histone positions referring to the top 9,841 regions (FDR 40 cut off) and found a total of 30,591 CpGs in our array. The large majority (98.9 %) of these CpGs were hypomethylated (n = 30,244) whereas only 1.1% of all histone-retained sites were hypermethylated (n = 347). Similarly to the globally considered “conserved” hypo/hypermethylated sites, we observed sharp differences in the distribution according to functional genomic and CpG content criteria between hypo and hypermethylated loci enriched in histones, since promoters and islands are prevalent in the hypomethylated loci (74% and 90.5%, respectively), and scarcely represented in the hypermethylated loci (22.8% and 40.3%, respectively) (Figure 7). Hypermethylated loci at the global level (including both histone-enriched and histone-depleted i.e. protaminized segments) have been found mainly outside of the islands as well as shore and shelf areas and involve 63.3% of the so called ”open sea/others” genomic regions whereas the same regions are present only in 21.6 % of hypermethylated histone-retained CpGs. Moreover, in hypermethylated loci overlapping with histones, islands were represented almost seven times more than in hypermethylated regions at the global level (40.3% versus 6.7%) (See Figure 6 and 7 ).

In agreement with Hammoud et al. [14] and Vavouri and Lenher [34], we also found that histone-retained hypomethylated regions were enriched with developmental genes (Table S4B) indicating that genes mapping to histone enriched regions are related to completely distinct biological processes compared to the hypomethylated region at the global level (i.e. involved in metabolism). Concerning histone enriched hypermethylated regions the level of associations was much lower with specific biological processes (below p<10^−4^) and was more heterogeneous. An additional datum supporting the link between hypomethylation and histone retention of developmental genes was that among the 106 developmental genes available in the array, 62 presented in their promoters CpGs with <20% of methylation level as well as histone retention. On the contrary, no overlap with histones was observed in developmental gene promoters showing hypermethylation.

#### Comparison of the sperm DNA methylome with a differentiated somatic cell type

**Figure 8:**
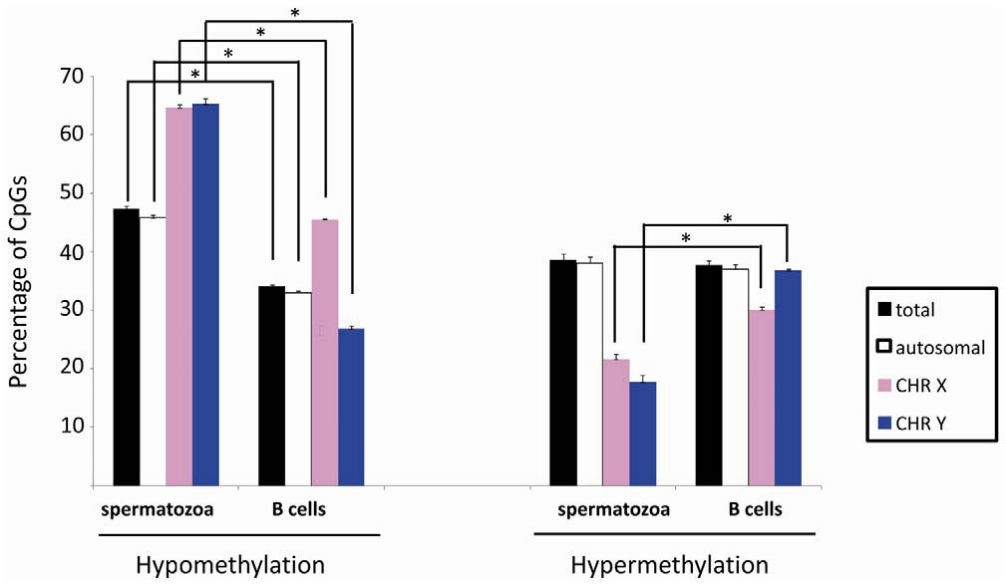
Bar graph illustrating the percentage of hypermethylated and hypomethylated CpGs in swim-up sperm samples and B cells. The number of detected CpGs varies according to the data extrapolation performed separately for each tested group: i) total CpGs, ii) autosomal CpGs, iii) X chromosome-linked CpGs and iv) Y chromosome-linked CpGs. * corresponds to p values <0.05 (the whiskers show the SD; n = 7).

**Figure 9:**
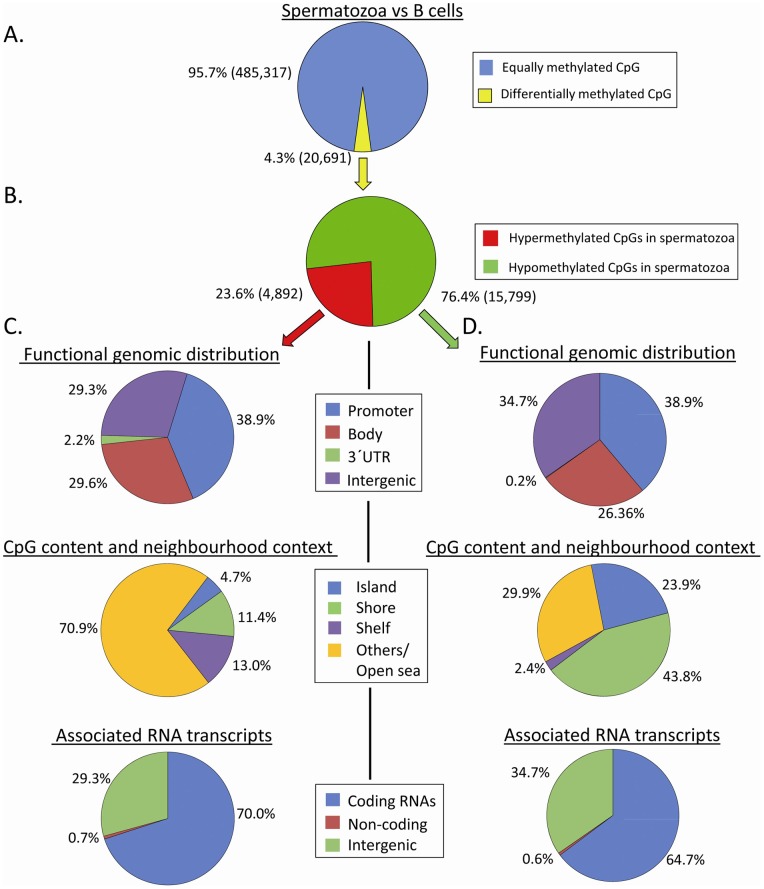
Spermatozoa versus B cell: a 450K DNA methylation portrait. (A) Graph showing percentages of equally and differentially methylated CpG sites in swim-up sperm samples compared to B cells. (B) Graph showing percentages of hypermethylation and hypomethylation in spermatozoa relating to the differentially methylated CpGs proportion (4,3%). Graphs describing the hypermethylated (C) and hypomethylated (D) sites according to their i) functional genomic distribution; ii) CpG content/neighborhood context and iii) association with RNA transcripts.

The average percentage of hypomethylated loci was significantly higher in sperm cells of the 8 subjects at the global, autosomal and sex-chromosomal levels (p<0.05 for all comparisons) compared to the differentiated somatic cell (Figure 8). On the contrary, no differences were found concerning the percentage of hypermethylated sites. The most striking difference in hypomethylation concerned the X and Y chromosomes (Figure 8 and Table 1). Next, we searched for the number of equally methylated CpGs between spermatozoa and B cells. We found that 4% showed a differentially methylated pattern whereas 485,317 CpGs were equally methylated between the two types of cells (96%). (Figure 9). Among the almost 20,000 CpGs showing a sperm-specific differentially methylated pattern (either hypo- or hypermethylation), those hypomethylated CpGs in spermatozoa, which were found to be hypermethylated in B cells, are the most represented proportion (76%). We analyzed the functional genomic distribution, CpG content and the associated RNA transcripts in the differentially methylated sites, separately for hypo-and hypermethylated sperm specific CpGs. The only significant difference consisted in the overrepresentation of “open sea/others” elements in the category of CpG content/neighborhood context of the sperm-specific hypermethylated CpGs compared to the sperm-specific hypomethylated sites (71% versus 30%, p = 0.00086). The 15,799 CpGs showing a sperm-specific hypomethylated pattern included 6,140 gene promoter CpGs which belong to 3,344 distinct genes. The function of these genes is mainly related to metabolic and biochemical processes, and among the strongest associations resulted also DNA methylation involved in gamete generation and piRNA metabolic processes (Table S5A). Furthermore, we identified a total of 195 genes with sperm-specific hypomethylated gene promoters which were associated with histone-retained regions and involved in developmental processes (organogenesis, especially neuronal development) and in spermatogenesis (Table S5B).

#### piRNAs and DNA methylation status

We were interested in providing a detailed description of the methylation status of piRNA-linked CpGs in spermatozoa, B cells and cancer cells. To accomplish this purpose, we crossed the position of 15,000 piRNAs with unique positions in the genome present in the piRNABank (http://pirnabank.ibab.ac.in/) with the positions of the 487,517 CpGs available in the array. In order to include potential regulatory sequences, we extended the piRNA positions to ±2 Kb and through these criteria a total of 2,591 unique piRNAs have been found to be covered by 7,528 CpGs on the array. In spermatozoa, 80% of the total array-available piRNA-linked CpGs (n = 6050) revealed either very high or very low methylation levels. In fact, similarly to the global sperm DNA methylome, sperm piRNA-linked CpGs showed a sharply polarized methylation profile being 48.6% hypomethylated (<20% methylation level) and 31.8% hypermethylated (>80% methylation level). We found a significantly higher proportion of piRNA-linked CpGs within the total hypomethylated loci (3,657 out of 220,300) compared to those found within the hypermethylated loci (2,392 out of 161,542 ) (p = 1.585E-05). In order to obtain more comprehensive characterization of sperm specific piRNAs-linked CpG methylation, we performed a comparative analysis with a differentiated somatic cell type (B cell) and a colon cancer cell type (HCT116).

By comparing the three cell types, we observed substantial differences in the methylation status of the piRNA-linked CpGs. In somatic cell, 95% of piRNA-linked CpGs show an intermediate methylation level, with a remaining 4.5% hypomethylated and 0.4% hypermethylated loci. On the contrary, similar to spermatozoa, cancer cells showed a polarization toward hypo/hypermethylation, but with an opposite pattern of methylation compared to spermatozoa i.e. 26% of the HCT116 cell piRNA-linked CpGs was hypomethylated and 53% was hypermethylated.

**Figure 10:**
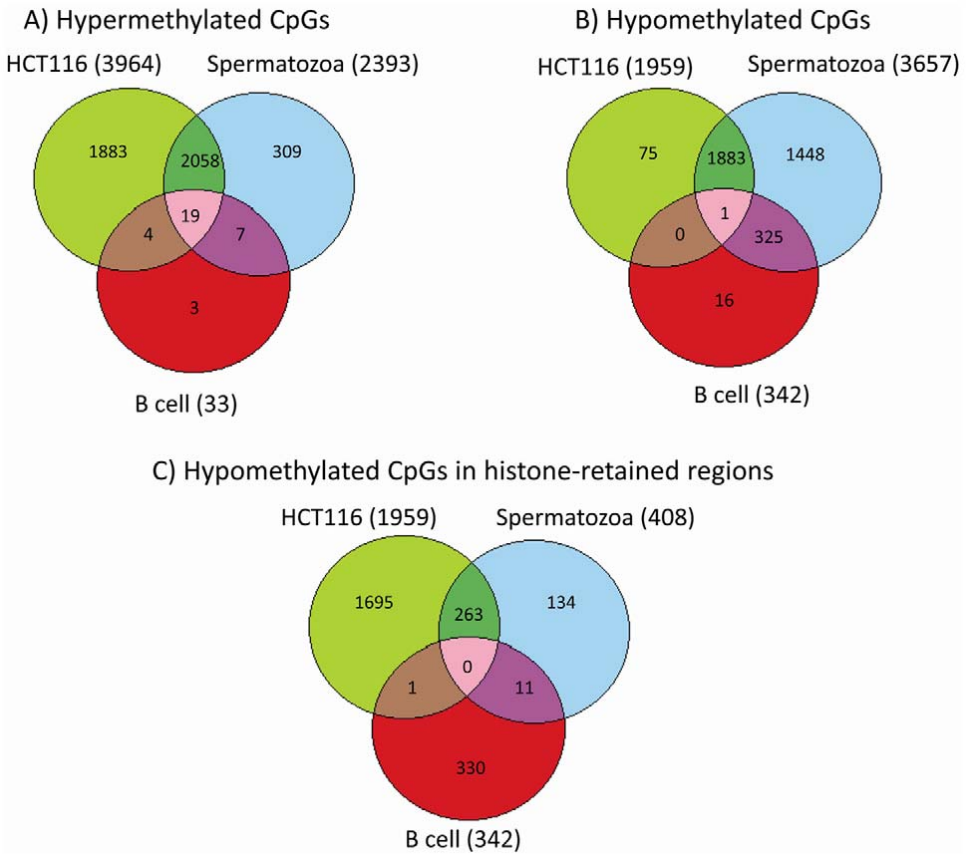
Venn diagram reporting unique and overlapping CpGs in/between the three cell types. A) Hypermethylated CpGs. B) Hypomethylated CpGs. C) Hypomethylated CpGs in histone-retained regions. HCT116: colorectal cancer cell line.

Next, we aimed to define the number of overlapping and distinct CpGs within the three cell types showing the same DNA methylation pattern (hypo or hypermethylation) ( Figure 10). Sperm DNA methylation profile largely overlaps with that of the cancer cell, especially for the hypermethylated loci (86.8%). The overlap was 51.5% within the hypomethylated CpGs. On the contrary, there is only a limited number of overlapping CpGs with the somatic cell, with the largest overlap within hypomethylated loci (8.9 %) and the lowest for the hypermethylated CpGs (1.1%). Given the functional importance of histone-retained regions in spermatozoa [14; [34], we extended our analysis to histone-retained regions associated to piRNAs A total of 408 piRNA-linked CpGs revealed to be overlapping with histone-retained regions in spermatozoa and interestingly, 97% of them showed <20% of methylation level. When comparing the 342 hypomethylated piRNA-linked CpGs in B cells to the 3,657 hypomethylated piRNA-linked CpGs in the sperm, we found that all, except 16 CpGs, were also present in spermatozoa. However, when comparing the same 342 hypomethylated piRNA-linked CpG sites in B cells to the 408 hypomethylated histone enriched piRNA-linked CpG sites in the sperm, only 3.2% of them overlapped. The same phenomenon was observed for the cancer cell i.e. almost all piRNA-linked hypomethylated loci in the HCT116 cell (1883 out of 1959) overlapped with the 3657 hypomethylated piRNA-linked CpGs in the sperm whereas only 263/1959 were shared between the two cell types when comparing to the sperm histone–enriched loci (408 CpG sites).

We next focused on the characterization of sperm-specific piRNAs. Accordingly, we identified the sperm-specific hypo and hypermethylated sites i.e. not overlapping with any of the two other cell types. We performed a GO analysis for the genes overlapping sperm-specific piRNA in order to define the type of biological processes in which the associated genes are involved (Table S6). A total of 213 genes were identified in association with piRNAs showing exclusive hypomethylation in the spermatozoa. Strikingly, some of these genes are involved in embryonic development.

## Discussion

The mammalian germ line undergoes extensive epigenetic reprogramming during development and gametogenesis. In pre-implantation embryo, a pattern of somatic-like DNA hypermethylation is established in all cells, including those which are destined to give origin to germ cells. This active *de novo* methylation process is followed by a widespread erasure of DNA methylation in primordial germ cells. Subsequently, another wave of *de novo* methylation takes place during spermatogenesis, ensuring a male germ line specific pattern of DNA methylation. The understanding of this complex process and the description of sperm DNA methylome have multiple implications, including evolutionary [15] and clinical aspects [35]. The entire sperm DNA methylome has been recently described by Molaro et al. [15] and it is based on the analysis of the whole semen (without quality fractioning) belonging to two sperm donors. Studies dealing with a larger group of subjects analyzed only a few genes or were based on low resolution arrays [16]–[19], [21]–[25]. This implies that information about what we can consider as a normal sperm DNA methylome and whether this methylome is stable among different normozoospermic individuals is still missing. We addressed the above questions by using the 450 K platform which allowed us to provide the most extensive and comprehensive investigation on DNA methylation profile, available to date, on quality fractioned sperm populations in a group of normozoospermic subjects. By comparing data from the whole sperm DNA methylome [15] with that obtained with our array, we found a highly significant correlation Rho = 0.97 (Figure S1), indicating that our data and conclusions are highly reliable.

Our first aim was to provide data on sperm DNA methylation profile in quality-fractioned spermatozoa from the same subjects. Human semen is peculiar for the heterogeneity of its sperm population presenting a number of different qualitative features that include kinetic, morphological, metabolic and genetic/chromatin differences. It is for this reason that sperm selection methods have been developed for assisted reproductive techniques in order to obtain a highly enriched subpopulation of spermatozoa exhibiting the best structural and functional characteristics, indicative of optimal fertilizing ability. The question whether these quality differences between sperm subpopulations are also reflecting modifications in the DNA methylation pattern has not been addressed so far. In fact, all studies published to date, except for one, focused on either whole semen or just one selected sperm subpopulation. Our analysis of 487,517 CpGs revealed a profound stability of the sperm DNA methylome without significant differences between sperm fractions enriched in “poor” (swim-down) and “good” quality (swim-up) spermatozoa. For all comparisons we obtained surprisingly high correlations (R^2^ >0.989) and the two subpopulations did not show distinct clustering of their methylation profiles. However, by analysing the epigenetic distances between the two sperm fractions we were able to detect a significant difference only in one subject, although the correlation between his two sperm subpopulations was high also in this case, R^2^ = 0.9896. We separately analyzed a series of genes for which DNA methylation defects had been previously reported in association with impaired sperm production/quality that included 45 imprinted genes, available on the array, and 10 additional genes selected from the literature. Despite expectation, the DNA methylation profile of these genes showed no differences in relationship with sperm quality. These data indicate that in normozoospermic men, the global DNA methylation profile is not affected significantly by structural and functional differences between sperm subpopulations. The extensive conservation of the DNA methylation status is especially surprising if we consider that differences have been described also at the metabolic level of “poor” quality spermatozoa which could theoretically influence the DNA methylation process [7], [8].

The definition of sperm DNA methylation profile between different normozoospermic subjects derives from a previous observation showing a significant epigenetic variability in human germ cells. Our aim was to further explore this observation both by increasing the number of analyzed CpGs (the previous study analyzed only12,198 CpG sites) and by comparing different sperm fractions from different normozoospermic individuals. Our data, clearly proved that the methylation pattern in different individuals showing similar sperm characteristics without contaminating cells is highly conserved. In fact, the discrepancy with the previous study may well be due to a technical issue i.e. to the presence of contaminating somatic cells, which could account for the observed inter-individual differences in the methylation profile. The highest correlation was found in the selected fraction enriched with “good” quality spermatozoa with R^2^ >0.98. This observation indicates that regardless of slight differences in life style factors, age and BMI, those cells which are designated to the fertilization process (good quality sperm-enriched fraction) show a highly stable sperm methylation profile between individuals. The few moderate smokers (< 10 cigarettes/day) included in the study did not cluster together, however it remains an important question whether sperm methylome can be altered by heavy smoking or other exogenous factors.

For the general description and comparative analyses of the sperm DNA methylome, we focused on the fraction enriched with “good” quality spermatozoa showing a complete lack of significant inter-individual differences. An interesting feature of the “normal” sperm DNA methylome is its highly polarized methylation profile towards the two extreme of DNA methylation levels: hypomethylation (<20%) and hypermethylation (>80%). We found that 96% of all CpGs belonged to one of the above categories. Hypo- and hypermethylated loci were highly conserved in different individuals reaching to a concordance of 95% for hypomethylated CpGs and to 83.3% for the hypermethylated ones. These, so called “conserved CpGs” were further analyzed in comparison with the relatively few “variable CpGs” (0.3%) present in spermatozoa and with the B cell DNA methylation profile. The qualitative analysis of hypo-, hyper- and variably methylated regions showed significant differences between the conserved hypomethylated loci and the other two methylation categories; in fact, we observed a significant enrichment with promoters (63.6%) and islands/shores -linked CpGs in the hypomethylated loci. On the other hand, among the hypermethylated and “variable” CpGs there was a significant overrepresentation of gene body-linked CpGs which, together with intergenic CpGs, build up >60% of all CpGs. The high inter-individual conservation of hypomethylated loci, especially abundant in promoter regions, suggests that normal spermatogenesis requires strictly controlled methylation levels in specific gene promoters. At this regard, for the first time we provide evidence about an exceptionally high number of “conserved” hypomethylated X and Y chromosome-linked loci which further supports previous predictions on the importance of sex chromosome linked genes in spermatogenesis and stimulates further research on the sex chromosome methylation status in pathological conditions [36] On the other hand, “variable” loci mainly in gene bodies and intergenic sequences may indicate their irrelevant role in spermatogenesis or may represent epigenetic changes which may act as fine-tuners of spermatogenetic efficiency and thus may contribute to the inter-individual variability of sperm production in normal healthy men.

An increasing number of studies are converging on the importance of histone-retained regions in spermatozoa for embryo development. The first study by Hammoud et al. [14] posited that genes involved in early embryonic development had a distinct chromatin status in sperm, being hypomethylated, histone-retained, enriched in H3K4me3 marks, and thus poised for expression. On the other hand, Brykczynska et al demonstrated that histone-retained regions with H3K27me3 mark may also play a role in post-fertilization, whereas histone H3Lys4 demethylation (H3K4m2) marks genes which are relevant in spermatogenesis [37]. In addition, an other recent study reports a striking link between the retention of nucleosomes in sperm and the establishment of DNA methylation-free regions in the early embryo [34]. By using the 450 K array, we found that “conserved” hypomethylated CpGs mapping inside histone-enriched regions were associated with genes involved in developmental processes. Accordingly, the majority of developmental gene promoters available in the array were mapping inside histone-retained regions. Interestingly, the correlation with developmental genes was missing when the entire set of “conserved” hypomethylated regions were analyzed. In fact, genes belonging to this category are, indeed, involved in metabolic processes which indicate a differential biological function of genes situated in histone-enriched and histone-depleted regions.

The most relevant finding concerning the comparison between the DNA methylation profiles of the male germ cell and the B cell, is that only a minority of CpGs showed differential methylation (4.6%) between the two cell types and was mainly due to the overrepresentation of hypomethylated loci in spermatozoa. A total of 3,344 distinct genes were related to sperm-specific hypomethylated CpGs and among the strongest associations appeared “DNA methylation involved in gamete generation” and “piRNA metabolic processes”. Similarly to the general sperm DNA methylome data, those genes (n = 195) which were hypomethylated in histone-retained regions were involved in developmental processes (organogenesis, especially neuronal development) and spermatogenesis. The different methylation, in respect to the somatic cell, of the promoters of spermatogenesis genes is in accordance with the well known importance of epigenetic regulation of cell specific functions. The association with developmental genes further reinforces the hypothesis about a programmed histone retention in spermatozoa, which would serve for rapid activation of genes involved in embryonic development.

Finally, the complete lack of studies focusing on the methylation status of piRNAs in spermatozoa prompted us to provide a detailed analysis of this specific class of small non coding RNAs. Although piRNAs were first described as specifically expressed in the testis, recent data suggest their potential role in tumorigenesis and in somatic cell function [29], [30]. In addition the presence of piRNAs has been also described in spermatozoa [38]. The 450K array is able to provide the characterization of a total of 2,591 unique piRNAs covered by 7,528 CpGs on the array. In spermatozoa we found a significantly higher proportion of piRNA-linked CpGs within the total hypomethylated loci compared to those found within the hypermethylated loci (p = 1.585E-05). The preferential hypomethylation of piRNAs was evident also in comparison with two other cell types: a differentiated somatic cell type (B cell) and a colon cancer cell type (HCT116). In fact, in spermatozoa 48.6% of CpGs were hypomethylated, whereas the percentages were 26% and 4.5% in HCT116 and B cell, respectively. Intriguingly, among those piRNAs which were located in histone-retained regions in spermatozoa, 97% of them showed low level of methylation. This observation represents a starting point for future studies aimed to explore the biological significance of these cell-dependent differences. An additional novel finding concerns the involvement of piRNA-related genes in distinct biological processes according to the methylation status of the related piRNAs. Most importantly, hypomethylated piRNAs are linked to genes associated with embryonic development and cell adhesion. Interestingly, piRNAs in histone-retained regions, showing hypomethylation exclusively in spermatozoa, are involved in the negative regulation of metabolic and biosynthetic processes which could be potentially relevant to the embryo. Given that almost all of these piRNAs are located inside or in the 3′UTR regions of the abovementioned genes, a potential RNA interfering mechanism can be hypothesized [29], [39]. The interference with those RNAs which would have a negative regulatory effect on metabolism and biosynthesis may have an important biological function in early embryonic development.

In conclusion, our study, based on the largest number of subjects ever considered for such a high number of CpGs, provided clear evidence of a highly conserved DNA methylation profile among normozoospermic subjects. We also demonstrated that sperm methylation is stable in different quality-fractioned sperm subpopulations of the same individual i.e. sperm methylation is not altered in “poor” quality spermatozoa of normozoospermic men despite the fact that these cells are clearly different from a metabolic and DNA integrity point of view. In addition, our array-based analysis provided both confirmatory and novel data concerning the “normal” sperm DNA methylome, including its peculiar features in respect to somatic and cancer cells. Our description about a highly polarized sperm methylation profile, the clearly distinct genomic and functional organization of hypo versus hypermethylated loci and the association of histone-enriched hypomethylated loci with embryonic development, which we now extended also to hypomethylated piRNAs-linked genes, represents a solid basis for future basic and clinically oriented research.

## Supporting Information

Figure S1 **Comparison of the methylation levels obtained in the array versus data reported in Molaro et** **al paper (ref 15).** Heatmap generated from the distance correlation matrix for the 8 individual samples “up” (A) and “down” (B), the scale of the correlation is shown above the matrix (scale values from 0 to 0.03); Correlation scatter plots between Molarós data vs the average methylation level for all “up” samples (C) and “down” samples (D), the Pearson correlation coefficient (rho) is shown.
(DOC)

Table S1 **Clinical description of the 8 normozoospermic individuals.**
(DOC)

Table S2 **Analysis of intra-and inter-individual variability of the sperm DNA methylation profile: estimate of correlation coefficients.**
(DOC)

Table S3 **Analysis of inter-individual variability of the sperm DNA methylation profile: epigenetic distance and coefficient of variation.**
(DOC)

Table S4 **Biological processes associated with genes linked to “conserved” hypo- and hypermethylated CpG loci in spermatozoa.**
(DOC)

Table S5 **Biological processes associated with genes linked to sperm-specific hypomethylated CpG loci.**
(DOC)

Table S6 **Biological processes associated with genes linked to piRNAs specifically hypo or hypermethylated in spermatozoa.**
(DOC)
