# Supplementary material for: Novel Insights into DNA Methylation Features in Spermatozoa: Stability and Peculiarities
Source: PLoS One. 2012 Oct 2;7(10):e44479. doi: 10.1371/journal.pone.0044479 (PMC3467000; doi:10.1371/journal.pone.0044479)
Supplement: Table S1 — Clinical description of the 8 normozoospermic individuals. (DOC) [file pone.0044479.s002.doc]

**Table S1. Clinical description of the 8 normozoospermic individuals.**

| **Sample** | **Age** | **BMI** | **Smoking** | **Total Sperm Number** | **Total Motile Sperm Number** |
| --- | --- | --- | --- | --- | --- |
| **EC01** | 33 | 24,18 | N | 735,9 | 404,8 |
| **EC07** | 35 | 23,08 | Y* | 1346,4 | 942,5 |
| **EC10** | 35 | 22,46 | Y* | 369,6 | 273,5 |
| **EC12** | 38 | 22,09 | Y* | 239,4 | 114,9 |
| **EC14** | 45 | 27,78 | N | 271,7 | 119,6 |
| **EC16** | 37 | 26,30 | Y* | 201,6 | 135,1 |
| **EC18** | 41 | 29,00 | N | 228 | 141,4 |
| **EC20** | 36 | 25,10 | Y* | 234,6 | 145,5 |
| **Mean ± DS** | 37.50± 3.85 | 25.00± 2.53 |  | 453.40 ± 400.92 | 284.64 ± 284.26 |

**Notes:** The age, Body Mass Index (BMI), smoking status as well as the total sperm number, the total motile sperm number, mean values ± DS for each parameter of the eight individuals are shown. N= No; Y*= individuals smoke <10 cigarettes/die

| **Sample** | **Up % Motility** | **Down % Motility** | **Up % Normal morphology** | **Down % Normal morphology** |
| --- | --- | --- | --- | --- |
| **EC01** | 91 | 55 | 25 | 15 |
| **EC07** | 85 | 70 | 20 | 14 |
| **EC10** | 89 | 71 | 14 | 7 |
| **EC12** | 88 | 48 | 15 | 7 |
| **EC14** | 89 | 47 | 20 | 8 |
| **EC16** | 94 | 67 | 20 | 8 |
| **EC18** | 94 | 62 | 15 | 9 |
| **EC20** | 96 | 62 | 19 | 12 |
| **Mean ± DS** | 90.75± 3.69 | 60.25± 9.38 | 18.5± 3.66 | 10.00± 3.21 |

**The % motility in the “Down” fraction refers to spermatozoa belonging to the “slow progressive + in situ” categories, whereas in the “Up” fraction spermatozoa showed “rapid or slow progressive” motility (no “in situ” motility was observed in the “up” fraction). Morphology has been scored according to the WHO manual 2010 edition.**
