# Supplementary material for: Novel Insights into DNA Methylation Features in Spermatozoa: Stability and Peculiarities
Source: PLoS One. 2012 Oct 2;7(10):e44479. doi: 10.1371/journal.pone.0044479 (PMC3467000; doi:10.1371/journal.pone.0044479)
Supplement: Table S2 — Analysis of intra-and inter-individual variability of the sperm DNA methylation profile: estimate of correlation coefficients. (DOC) [file pone.0044479.s003.doc]

**Table S2. Analysis of intra-and inter-individual variability of the sperm DNA methylation profile**.

| **A. Correlation Coefficient (R^2^)** | |
| --- | --- |
| Code | Up vs Dn |
| EC 01 | 0.9977 |
| EC 07 | na |
| EC 10 | 0.998 |
| EC 12 | 0.9896 |
| EC 14 | 0.998 |
| EC 16 | 0.9975 |
| EC 18 | 0.9964 |
| EC 20 | 0.9982 |

| **B. Correlation Coefficient (R^2^)** | |
| --- | --- |
| **Code** | **Up** |
| EC1 vs EC7 | 0.9864 |
| EC1 vs EC10 | 0.9887 |
| EC1 vs EC12 | 0.9875 |
| EC1 vs EC14 | 0.9875 |
| EC1 vs EC16 | 0.9881 |
| EC1 vs EC18 | 0.9817 |
| EC1 vs EC20 | 0.9883 |
| EC7 vs EC10 | 0.9851 |
| EC7 vs EC12 | 0.9873 |
| EC7 vs EC14 | 0.9821 |
| EC7 vs EC16 | 0.9833 |
| EC7 vs EC18 | 0.9836 |
| EC7 vs EC20 | 0.9867 |
| EC10 vs EC12 | 0.9883 |
| EC10 vs EC14 | 0.9863 |
| EC10 vs EC16 | 0.9864 |
| EC10 vs EC18 | 0.9829 |
| EC10 vs EC20 | 0.9887 |
| EC12 vs EC14 | 0.9857 |
| EC12 vs EC16 | 0.9849 |
| EC12 vs EC18 | 0.9856 |
| EC12 vs EC20 | 0.9887 |
| EC14 vs EC16 | 0.9849 |
| EC14 vs EC18 | 0.9818 |
| EC14 vs EC20 | 0.9856 |
| EC16 vs EC18 | 0.979 |
| EC16 vs EC20 | 0.9881 |
| EC18 vs EC20 | 0.9847 |

**Notes**: Pearson correlation coefficients (R^2^) observed when analyzing: A) Intra-individual variability through the comparison of swim-up (Up) versus swim-down (Dn) sperm fractions in the same individual; B) Inter-individual variability through the comparison of swim-up (Up) sperm fractions between different individuals.
