# Supplementary material for: Novel Insights into DNA Methylation Features in Spermatozoa: Stability and Peculiarities
Source: PLoS One. 2012 Oct 2;7(10):e44479. doi: 10.1371/journal.pone.0044479 (PMC3467000; doi:10.1371/journal.pone.0044479)
Supplement: Table S3 — Analysis of inter-individual variability of the sperm DNA methylation profile: epigenetic distance and coefficient of variation. (DOC) [file pone.0044479.s004.doc]

**Table S3. Analysis of inter-individual variability of the sperm DNA methylation profile.**

| **A. Inter-individual epigenetic distances per group** | | | | | |
| --- | --- | --- | --- | --- | --- |
| **Ws 1h** | | **Up** | | **Dn** | |
| EC01vsEC02 | 31.90 | EC01vsEC02 | 31.72 | EC01vsEC02 | 31.63 |
| EC01vsEC03 | 29.14 | EC01vsEC03 | 28.48 | EC01vsEC03 | 40.43 |
| EC01vsEC04 | 29.98 | EC01vsEC04 | 29.87 | EC01vsEC04 | 30.71 |
| EC01vsEC05 | 30.37 | EC01vsEC05 | 29.23 | EC01vsEC05 | 34.10 |
| EC01vsEC06 | 29.95 | EC01vsEC06 | 29.04 | EC01vsEC06 | 35.72 |
| EC01vsEC07 | 36.06 | EC01vsEC07 | 37.77 | EC01vsEC07 | 30.61 |
| EC01vsEC08 | 29.99 | EC01vsEC08 | 28.85 | EC02vsEC03 | 41.28 |
| EC02vsEC03 | 33.04 | EC02vsEC03 | 33.23 | EC02vsEC04 | 31.77 |
| EC02vsEC04 | 30.89 | EC02vsEC04 | 30.55 | EC02vsEC05 | 30.85 |
| EC02vsEC05 | 37.97 | EC02vsEC05 | 36.21 | EC02vsEC06 | 34.34 |
| EC02vsEC06 | 35.52 | EC02vsEC06 | 35.27 | EC02vsEC07 | 28.54 |
| EC02vsEC07 | 35.55 | EC02vsEC07 | 35.76 | EC03vsEC04 | 45.41 |
| EC02vsEC08 | 32.34 | EC02vsEC08 | 31.33 | EC03vsEC05 | 44.72 |
| EC03vsEC04 | 29.14 | EC03vsEC04 | 29.04 | EC03vsEC06 | 39.05 |
| EC03vsEC05 | 32.66 | EC03vsEC05 | 31.70 | EC03vsEC07 | 40.08 |
| EC03vsEC06 | 31.74 | EC03vsEC06 | 31.26 | EC04vsEC05 | 33.96 |
| EC03vsEC07 | 35.96 | EC03vsEC07 | 37.10 | EC04vsEC06 | 37.09 |
| EC03vsEC08 | 30.17 | EC03vsEC08 | 28.55 | EC04vsEC07 | 32.21 |
| EC04vsEC05 | 31.90 | EC04vsEC05 | 32.06 | EC05vsEC06 | 38.53 |
| EC04vsEC06 | 31.66 | EC04vsEC06 | 32.60 | EC05vsEC07 | 29.84 |
| EC04vsEC07 | 34.59 | EC04vsEC07 | 33.35 | EC06vsEC07 | 31.91 |
| EC04vsEC08 | 28.96 | EC04vsEC08 | 28.34 |  |  |
| EC05vsEC06 | 32.11 | EC05vsEC06 | 33.06 |  |  |
| EC05vsEC07 | 39.88 | EC05vsEC07 | 36.86 |  |  |
| EC05vsEC08 | 32.66 | EC05vsEC08 | 32.15 |  |  |
| EC06vsEC07 | 40.18 | EC06vsEC07 | 40.29 |  |  |
| EC06vsEC08 | 30.03 | EC06vsEC08 | 29.08 |  |  |
| EC07vsEC08 | 33.95 | EC07vsEC08 | 34.55 |  |  |

| **B. Coefficient of variation per group** | | |
| --- | --- | --- |
| **Ws 1h** | **Up** | **Dn** |
| 9.57% | 10.12% | 14.04% |

**Notes:** The inter-individual variability was estimated in terms of: A) Epigenetic distances (measured with the Euclidean formula) between different individuals and B) Coefficient of variation among individuals.Ws: Whole sperm population; Up: swim-up fraction; Dn: swim-down fraction
