# Supplementary material for: Novel Insights into DNA Methylation Features in Spermatozoa: Stability and Peculiarities
Source: PLoS One. 2012 Oct 2;7(10):e44479. doi: 10.1371/journal.pone.0044479 (PMC3467000; doi:10.1371/journal.pone.0044479)
Supplement: Table S4 — Biological processes associated with genes linked to “conserved” hypo- and hypermethylated CpG loci in spermatozoa. (DOC) [file pone.0044479.s005.doc]

**Table S4. Biological processes significantly associated with genes linked to “conserved” hypomethylated and hypermethylated CpGs.**

| **A1. Total CpGs: Hypomethylation** | | | | |
| --- | --- | --- | --- | --- |
| **Biological Process** | **p value** | **FDR** | **GOBPID** | **OddsRatio** |
| cellular process | 7.03E-175 | 1.41E-173 | GO:0051716 | 4.06 |
| cellular macromolecule metabolic process | 7.08E-146 | 7.08E-145 | GO:0007165 | 3.06 |
| cellular metabolic process | 1.41E-135 | 9.40E-135 | GO:0023052 | 2.73 |
| Nucleoside, nucleotide and nucleic acid metabolic process | 5.51E-110 | 2.76E-109 | GO:0006811 | 2.86 |
| nucleic acid metabolic process | 6.81E-104 | 2.72E-103 | GO:0051234 | 3.00 |
| cellular nitrogen compound metabolic process | 9.77E-103 | 3.26E-102 | GO:0006810 | 2.65 |
| cellular macromolecule biosynthetic process | 9.87E-101 | 2.82E-100 | GO:0051179 | 3.05 |
| nitrogen compound metabolic process | 1.37E-96 | 3.43E-96 | GO:0043412 | 2.54 |
| macromolecule biosynthetic process | 5.11E-95 | 1.14E-94 | GO:0006464 | 2.9 |
| cellular biosynthetic process | 2.84E-94 | 5.68E-94 | GO:0006812 | 2.64 |
| gene expression | 3.41E-94 | 6.20E-94 | GO:0035556 | 2.84 |
| biosynthetic process | 7.04E-92 | 1.17E-91 | GO:0030001 | 2.58 |
| metabolic process | 8.48E-92 | 1.30E-91 | GO:0050896 | 2.27 |
| primary metabolic process | 1.75E-91 | 2.50E-91 | GO:0006468 | 2.26 |
| macromolecule metabolic process | 9.08E-91 | 1.21E-90 | GO:0032879 | 2.31 |
| regulation of cellular macromolecule biosynthetic process | 1.54E-75 | 1.93E-75 | GO:0055085 | 2.85 |
| transcription | 1.07E-74 | 1.26E-74 | GO:0007166 | 2.84 |
| regulation of gene expression | 1.55E-70 | 1.72E-70 | GO:0065008 | 2.68 |
| RNA metabolic process | 3.77E-70 | 3.97E-70 | GO:0007169 | 2.76 |
| regulation of cellular metabolic process | 3.97E-70 | 3.97E-70 | GO:0071702 | 2.39 |

| **A2. Total CpGs: Hypermethylation** | | | | |
| --- | --- | --- | --- | --- |
| **Biological Process** | **Pvalue** | **FDR** | **GOBPID** | **OddsRatio** |
| cellular response to stimulus | 3.59E-23 | 7.18E-22 | GO:0009987 | 1.62 |
| signal transduction | 3.84E-21 | 3.84E-20 | GO:0044260 | 1.63 |
| signaling | 6.03E-21 | 4.02E-20 | GO:0044237 | 1.59 |
| ion transport | 6.85E-19 | 3.43E-18 | GO:0006139 | 2.54 |
| establishment of localization | 1.81E-18 | 7.24E-18 | GO:0090304 | 1.61 |
| transport | 1.07E-17 | 3.57E-17 | GO:0034641 | 1.59 |
| localization | 1.50E-17 | 4.29E-17 | GO:0034645 | 1.54 |
| macromolecule modification | 1.49E-13 | 3.73E-13 | GO:0006807 | 1.59 |
| protein modification process | 3.51E-13 | 7.80E-13 | GO:0009059 | 1.59 |
| cation transport | 9.40E-13 | 1.88E-12 | GO:0044249 | 2.44 |
| intracellular signal transduction | 5.95E-12 | 1.08E-11 | GO:0010467 | 1.65 |
| metal ion transport | 1.40E-11 | 2.33E-11 | GO:0009058 | 2.47 |
| response to stimulus | 1.80E-10 | 2.77E-10 | GO:0008152 | 1.31 |
| protein phosphorylation | 3.67E-10 | 4.63E-10 | GO:0044238 | 1.76 |
| regulation of localization | 3.70E-10 | 4.63E-10 | GO:0043170 | 1.84 |
| transmembrane transport | 3.70E-10 | 4.63E-10 | GO:2000112 | 1.84 |
| cell surface receptor linked signaling pathway | 4.13E-10 | 4.86E-10 | GO:0006350 | 1.51 |
| regulation of biological quality | 1.05E-09 | 1.17E-09 | GO:0010468 | 1.46 |
| transmembrane receptor tyrosine kinase signaling pathway | 3.43E-09 | 3.61E-09 | GO:0016070 | 2.13 |
| organic substance transport | 4.25E-09 | 4.25E-09 | GO:0031323 | 2.24 |

| **B1. CpGs associated with histone-retained regions: Hypomethylation** | | | | |
| --- | --- | --- | --- | --- |
| **Biological Process** | **p value** | **FDR** | **GOBPID** | **OddsRatio** |
| nervous system development | 1.23E-39 | 2.34E-38 | GO:0007399 | 2.32 |
| multicellular organismal development | 2.75E-39 | 2.61E-38 | GO:0007275 | 1.86 |
| developmental process | 1.72E-38 | 1.09E-37 | GO:0032502 | 1.82 |
| anatomical structure development | 8.78E-38 | 4.17E-37 | GO:0048856 | 1.86 |
| system development | 4.91E-37 | 1.87E-36 | GO:0048731 | 1.89 |
| anatomical structure morphogenesis | 4.08E-36 | 1.29E-35 | GO:0009653 | 2.18 |
| cell differentiation | 1.11E-35 | 3.01E-35 | GO:0030154 | 1.99 |
| cellular developmental process | 1.08E-33 | 2.57E-33 | GO:0048869 | 1.94 |
| neurogenesis | 2.67E-32 | 5.64E-32 | GO:0022008 | 2.52 |
| generation of neurons | 3.04E-31 | 5.78E-31 | GO:0048699 | 2.54 |
| neuron differentiation | 1.67E-30 | 2.88E-30 | GO:0030182 | 2.57 |
| cell development | 2.80E-29 | 4.43E-29 | GO:0048468 | 2.26 |
| regulation of cellular process | 1.32E-28 | 1.93E-28 | GO:0050794 | 1.6 |
| regulation of biological process | 2.15E-25 | 2.92E-25 | GO:0050789 | 1.55 |
| biological regulation | 9.18E-25 | 1.16E-24 | GO:0065007 | 1.55 |
| embryo development | 6.31E-23 | 7.49E-23 | GO:0009790 | 2.39 |
| organ morphogenesis | 7.38E-23 | 8.25E-23 | GO:0009887 | 2.5 |
| brain development | 2.05E-22 | 2.16E-22 | GO:0007420 | 3.1 |
| signaling | 2.79E-22 | 2.79E-22 | GO:0023052 | 1.56 |
| central nervous system development | 2.98E-22 | 3.02E-21 | GO:0007417 | 2.64 |

| **B2. CpGs associated with histone-retained regions: Hypermethylation** | | | | |
| --- | --- | --- | --- | --- |
| **Biological Process** | **Pvalue** | **FDR** | **GOBPID** | **OddsRatio** |
| serotonin secretion | 1.54E-04 | 0.00062 | GO:0001820 | 172.92 |
| cerebellar granular layer development | 1.54E-04 | 0.00062 | GO:0021681 | 172.92 |
| cerebellar granular layer morphogenesis | 1.54E-04 | 0.00062 | GO:0021683 | 172.92 |
| cerebellar granular layer formation | 1.54E-04 | 0.00062 | GO:0021684 | 172.92 |
| cerebellar granule cell differentiation | 1.54E-04 | 0.00062 | GO:0021707 | 172.92 |
| serotonin transport | 3.22E-04 | 0.00107 | GO:0006837 | 103.74 |
| cerebellar cortex formation | 1.00E-03 | 0.00235 | GO:0021697 | 51.85 |
| cell projection morphogenesis | 1.16E-03 | 0.00235 | GO:0048858 | 4.27 |
| cell part morphogenesis | 1.21E-03 | 0.00235 | GO:0032990 | 4.23 |
| in utero embryonic development | 1.22E-03 | 0.00235 | GO:0001701 | 6.86 |
| cell differentiation in hindbrain | 1.37E-03 | 0.00235 | GO:0021533 | 43.2 |
| cell morphogenesis involved in differentiation | 1.41E-03 | 0.00235 | GO:0000904 | 4.13 |
| cell projection organization | 1.57E-03 | 0.00242 | GO:0030030 | 3.71 |
| cerebellar cortex morphogenesis | 1.80E-03 | 0.00257 | GO:0021696 | 37.03 |
| cell morphogenesis involved in neuron differentiation | 2.82E-03 | 0.00340 | GO:0048667 | 4.08 |
| cerebellar cortex development | 2.82E-03 | 0.00340 | GO:0021695 | 28.79 |
| neuron projection morphogenesis | 2.89E-03 | 0.00340 | GO:0048812 | 4.06 |
| cerebellum morphogenesis | 3.11E-03 | 0.00346 | GO:0021587 | 27.27 |
| cell morphogenesis | 3.43E-03 | 0.00361 | GO:0000902 | 3.55 |
| regulation of skeletal muscle fiber development | 4.05E-03 | 0.00405 | GO:0048742 | 23.55 |

**Notes:** The upper table (A) refers to all CpGs with “conserved” methylation level among the eight individuals, while the lower table (B) relates to the portion of “conserved” CpGs overlapping with histones. Both refer to swim-up sperm fractions. Only the first 20 most significant items are shown.
