# Supplementary material for: Novel Insights into DNA Methylation Features in Spermatozoa: Stability and Peculiarities
Source: PLoS One. 2012 Oct 2;7(10):e44479. doi: 10.1371/journal.pone.0044479 (PMC3467000; doi:10.1371/journal.pone.0044479)
Supplement: Table S5 — Biological processes associated with genes linked to sperm-specific hypomethylated CpG loci. (DOC) [file pone.0044479.s006.doc]

**Table S5. List of biological processes significantly associated with genes linked to sperm-specific**

**hypomethylated CpG loci.**

| **A. Sperm specific hypomethylated CpGs** | | | | |
| --- | --- | --- | --- | --- |
| **Biological Process** | **p value** | **FDR** | **GOBPID** | **OddsRatio** |
| cellular macromolecule metabolic process | 1.46E-09 | 1.68E-07 | GO:0044260 | 1.31 |
| cellular metabolic process | 1.58E-08 | 9.09E-07 | GO:0044237 | 1.28 |
| nitrogen compound metabolic process | 4.23E-08 | 1.63E-06 | GO:0006807 | 1.28 |
| nucleobase, nucleoside, nucleotide and nucleic acid metabolic process | 9.64E-08 | 2.77E-06 | GO:0006139 | 1.28 |
| nucleic acid metabolic process | 1.79E-07 | 3.45E-06 | GO:0090304 | 1.28 |
| cellular nitrogen compound metabolic process | 1.80E-07 | 3.45E-06 | GO:0034641 | 1.26 |
| DNA methylation involved in gamete generation | 5.70E-07 | 9.37E-06 | GO:0043046 | 47.54 |
| cellular macromolecule biosynthetic process | 4.08E-06 | 5.61E-05 | GO:0034645 | 1.25 |
| cellular process | 4.39E-06 | 5.61E-05 | GO:0009987 | 1.34 |
| macromolecule metabolic process | 6.11E-06 | 7.03E-05 | GO:0043170 | 1.22 |
| gene expression | 6.98E-06 | 7.30E-05 | GO:0010467 | 1.24 |
| macromolecule biosynthetic process | 8.83E-06 | 8.46E-05 | GO:0009059 | 1.24 |
| metabolic process | 1.80E-05 | 0.000149 | GO:0008152 | 1.21 |
| piRNA metabolic process | 1.80E-05 | 0.000149 | GO:0034587 | 36.94 |
| cell cycle phase | 2.89E-05 | 0.000210 | GO:0022403 | 1.48 |
| cellular biosynthetic process | 2.91E-05 | 0.000210 | GO:0044249 | 1.21 |
| biosynthetic process | 3.38E-05 | 0.000229 | GO:0009058 | 1.21 |
| primary metabolic process | 6.06E-05 | 0.000387 | GO:0044238 | 1.19 |
| cell cycle | 6.49E-05 | 0.000393 | GO:0007049 | 1.35 |
| cell cycle process | 7.55E-05 | 0.000434 | GO:0022402 | 1.4 |

| **B. Sperm specific hypomethylated CpGs with histone retention** | | | | |
| --- | --- | --- | --- | --- |
| **Biological Process** | **Pvalue** | **FDR** | **GOBPID** | **OddsRatio** |
| signaling pathway involved in ventral spinal cord interneuron specification | 0.00009 | 0.00162 | GO:0021775 | Inf |
| smoothened signaling pathway involved in spinal cord motor neuron cell fate | 0.00009 | 0.00162 | GO:0021776 | Inf |
| signaling pathway involved in dorsal/ventral neural tube patterning | 0.00028 | 0.00271 | GO:0060831 | 208 |
| organ formation | 0.00031 | 0.00271 | GO:0048645 | 14.03 |
| smoothened signaling pathway involved in ventral spinal cord patterning | 0.00055 | 0.00380 | GO:0021910 | 104 |
| regulation of small GTPase mediated signal transduction | 0.00065 | 0.00380 | GO:0051056 | 3.48 |
| small GTPase mediated signal transduction | 0.00080 | 0.00401 | GO:0007264 | 3.03 |
| Rho protein signal transduction | 0.00113 | 0.00475 | GO:0007266 | 5.54 |
| hindgut morphogenesis | 0.00136 | 0.00475 | GO:0007442 | 51.95 |
| ventral spinal cord interneuron specification | 0.00136 | 0.00475 | GO:0021521 | 51.95 |
| ventral spinal cord interneuron differentiation | 0.00189 | 0.00508 | GO:0021514 | 41.55 |
| spinal cord motor neuron cell fate specification | 0.00189 | 0.00508 | GO:0021520 | 41.55 |
| ventral spinal cord interneuron fate commitment | 0.00189 | 0.00508 | GO:0060579 | 41.55 |
| negative regulation of organic acid transport | 0.00250 | 0.00625 | GO:0032891 | 34.63 |
| positive regulation of translational initiation | 0.00320 | 0.00746 | GO:0045948 | 29.68 |
| regulation of Rho protein signal transduction | 0.00444 | 0.00843 | GO:0035023 | 4.97 |
| regulation of insulin secretion | 0.00461 | 0.00843 | GO:0050796 | 4.93 |
| spermatogenesis | 0.00479 | 0.00843 | GO:0007283 | 3.00 |
| male gamete generation | 0.00479 | 0.00843 | GO:0048232 | 3.00 |
| cranial nerve morphogenesis | 0.00482 | 0.00843 | GO:0021602 | 23.08 |

**Notes:** The upper table (A) refers to all CpGs showing sperm-specific hypomethylation level, while the lower table (B) relates to the portion of hypomethylated CpGs mapping in histone-retained regions. Only the first 20 most significant items are shown.
