# Supplementary material for: Novel Insights into DNA Methylation Features in Spermatozoa: Stability and Peculiarities
Source: PLoS One. 2012 Oct 2;7(10):e44479. doi: 10.1371/journal.pone.0044479 (PMC3467000; doi:10.1371/journal.pone.0044479)
Supplement: Table S6 — Biological processes associated with genes linked to piRNAs specifically hypo or hypermethylated in spermatozoa. (DOC) [file pone.0044479.s007.doc]

**Table S6. Biological processes associated with genes linked to piRNAs specifically hypo- or hypermethylated in spermatozoa.**

| **A. Genes associated with piRNAs hypomethylated exclusively in spermatozoa** | | | | |
| --- | --- | --- | --- | --- |
| **Biological Process** | **p value** | **FDR** | **GOBPID** | **OddsRatio** |
| homophilic cell adhesion | 1.22E-27 | 3.54E-26 | GO:0007156 | 29.27 |
| cell-cell adhesion | 2.74E-22 | 3.97E-21 | GO:0016337 | 13.46 |
| cell adhesion | 2.07E-11 | 1.50E-10 | GO:0007155 | 4.87 |
| biological adhesion | 2.07E-11 | 1.50E-10 | GO:0022610 | 4.87 |
| embryonic skeletal system development | 1.37E-06 | 7.95E-06 | GO:0048706 | 11.49 |
| calcium-dependent cell-cell adhesion | 5.04E-06 | 2.43E-05 | GO:0016339 | 24.52 |
| embryonic skeletal system morphogenesis | 2.80E-05 | 1.16E-04 | GO:0048704 | 11.50 |
| regionalization | 5.88E-04 | 2.13E-03 | GO:0003002 | 4.14 |
| embryonic organ morphogenesis | 9.42E-04 | 2.74E-03 | GO:0048562 | 4.87 |
| skeletal system development | 9.46E-04 | 2.74E-03 | GO:0001501 | 3.55 |
| anterior/posterior pattern formation | 1.09E-03 | 2.77E-03 | GO:0009952 | 4.74 |
| assembly of spliceosomal tri-snRNP | 1.43E-03 | 2.77E-03 | GO:0000244 | 50.48 |
| negative regulation of adaptive immune response | 1.43E-03 | 2.77E-03 | GO:0002820 | 50.48 |
| negative regulation of adaptive immune response …. | 1.43E-03 | 2.77E-03 | GO:0002823 | 50.48 |
| neuron cell-cell adhesion | 1.43E-03 | 2.77E-03 | GO:0007158 | 50.48 |
| multicellular organismal development | 1.64E-03 | 2.97E-03 | GO:0007275 | 1.74 |
| skeletal system morphogenesis | 1.75E-03 | 2.98E-03 | GO:0048705 | 5.07 |
| negative regulation of leukocyte mediated immunity | 2.64E-03 | 3.83E-03 | GO:0002704 | 33.65 |
| negative regulation of lymphocyte mediated immunity | 2.64E-03 | 3.83E-03 | GO:0002707 | 33.65 |
| definitive hemopoiesis | 2.64E-03 | 3.83E-03 | GO:0060216 | 33.65 |

| **B. Genes associated with piRNAs hypermethylated exclusively in spermatozoa** | | | | |
| --- | --- | --- | --- | --- |
| **Biological Process** | **p value** | **FDR** | **GOBPID** | **OddsRatio** |
| anterograde axon cargo transport | 7.40E-05 | 0.00185 | GO:0008089 | 234.66 |
| axon cargo transport | 4.44E-04 | 0.00555 | GO:0008088 | 78.18 |
| regulation of excitatory postsynaptic membrane potential | 9.19E-04 | 0.00754 | GO:0060079 | 52.10 |
| regulation of postsynaptic membrane potential | 1.33E-03 | 0.00754 | GO:0060078 | 42.61 |
| microtubule-based transport | 1.81E-03 | 0.00754 | GO:0010970 | 36.05 |
| cytoskeleton-dependent intracellular transport | 1.81E-03 | 0.00754 | GO:0030705 | 36.05 |
| synaptic transmission | 4.08E-03 | 0.00814 | GO:0007268 | 5.33 |
| microtubule-based process | 4.65E-03 | 0.00814 | GO:0007017 | 6.58 |
| membrane depolarization | 5.25E-03 | 0.00814 | GO:0051899 | 20.35 |
| transmission of nerve impulse | 6.56E-03 | 0.00814 | GO:0019226 | 4.73 |
| multicellular organismal signaling | 6.56E-03 | 0.00814 | GO:0035637 | 4.73 |
| nerve-nerve synaptic transmission | 6.84E-03 | 0.00814 | GO:0007270 | 17.65 |
| response to calcium ion | 8.89E-03 | 0.00902 | GO:0051592 | 15.33 |

| **C. Genes associated with piRNAs hypomethylated specifically in spermatozoa vs cancer cell in histone-retained regions** | | | | |
| --- | --- | --- | --- | --- |
| **Biological Process** | **p value** | **FDR** | **GOBPID** | **OddsRatio** |
| negative regulation of nucleobase, nucleoside, nucleotide and nucleic acid metabolic process | 0.00422 | 0.00952 | GO:0045934 | 5.54 |
| negative regulation of nitrogen compound metabolic process | 0.00439 | 0.00952 | GO:0051172 | 5.49 |
| negative regulation of cellular biosynthetic process | 0.00585 | 0.00952 | GO:0031327 | 5.11 |
| negative regulation of biosynthetic process | 0.00626 | 0.00952 | GO:0009890 | 5.02 |
| G-protein coupled receptor protein signaling pathway | 0.00952 | 0.00952 | GO:0007186 | 5.52 |

| **D. Genes associated with piRNAs hypomethylated specifically in spermatozoa vs B cell in histone-retained regions** | | | | |
| --- | --- | --- | --- | --- |
| **Biological Process** | **p value** | **FDR** | **GOBPID** | **OddsRatio** |
| cellular macromolecule biosynthetic process | 8.83E-05 | 0.00190 | GO:0034645 | 2.35 |
| macromolecule biosynthetic process | 1.34E-04 | 0.00190 | GO:0009059 | 2.30 |
| biosynthetic process | 1.41E-04 | 0.00190 | GO:0009058 | 2.25 |
| cellular biosynthetic process | 1.85E-04 | 0.00190 | GO:0044249 | 2.22 |
| negative regulation of epithelial cell proliferation involved in prostate gland development | 5.19E-04 | 0.00426 | GO:0060770 | 85.26 |
| gene expression | 1.21E-03 | 0.00615 | GO:0010467 | 2.01 |
| regulation of epithelial cell proliferation involved in prostate gland development | 1.23E-03 | 0.00615 | GO:0060768 | 48.71 |
| epithelial cell proliferation involved in prostate gland development | 1.53E-03 | 0.00615 | GO:0060767 | 42.62 |
| glycoprotein biosynthetic process | 1.63E-03 | 0.00615 | GO:0009101 | 5.16 |
| regulation of gene expression | 1.67E-03 | 0.00615 | GO:0010468 | 2.03 |
| regulation of cellular macromolecule biosynthetic process | 1.93E-03 | 0.00615 | GO:2000112 | 2.03 |
| regulation of macromolecule metabolic process | 2.53E-03 | 0.00615 | GO:0060255 | 1.93 |
| regulation of macromolecule biosynthetic process | 2.61E-03 | 0.00615 | GO:0010556 | 1.98 |
| negative regulation of cellular biosynthetic process | 2.68E-03 | 0.00615 | GO:0031327 | 2.79 |
| regulation of nucleobase, nucleoside, nucleotide and nucleic acid metabolic process | 2.69E-03 | 0.00615 | GO:0019219 | 1.96 |
| regulation of cellular biosynthetic process | 2.76E-03 | 0.00615 | GO:0031326 | 1.96 |
| regulation of primary metabolic process | 2.84E-03 | 0.00615 | GO:0080090 | 1.90 |
| negative regulation of biosynthetic process | 3.06E-03 | 0.00615 | GO:0009890 | 2.74 |
| regulation of biosynthetic process | 3.08E-03 | 0.00615 | GO:0009889 | 1.94 |

**Notes:** Biological processes significantly associated with genes linked to piRNAs: (A) piRNAs showing hypomethylation exclusively in spermatozoa in respect to both B cell and HCT-116 cell line; (B) piRNAs showing hypermethylation exclusively in spermatozoa in respect to both B cell and HCT116 cell line; (C) piRNAs hypomethylated specifically in spermatozoa vs HCT116 cell line in histone-retained regions. (D) piRNAs hypomethylated specifically in spermatozoa vs B cell in histone-retained regions.
